# Exploiting the Synergy between Carboplatin and ABT-737 in the Treatment of Ovarian Carcinomas

**DOI:** 10.1371/journal.pone.0081582

**Published:** 2014-01-06

**Authors:** Harsh Vardhan Jain, Alan Richardson, Michael Meyer-Hermann, Helen M. Byrne

**Affiliations:** 1 Department of Mathematics, Florida State University, Tallahassee, Florida,United States of America; 2 Institute for Science and Technology in Medicine, Keele University, Stoke-on-Trent, United Kingdom; 3 Department of Systems Immunology, Helmholtz Centre for Infection Research, Braunschweig, Germany; 4 Bio Centre for Life Science, Braunschweig University of Technology, Braunschweig, Germany; 5 Oxford Centre for Collaborative and Applied Mathematics, Mathematical Institute, University of Oxford, Oxford, United Kingdom; 6 Department of Computer Science, University of Oxford, Oxford, United Kingdom; University of California-Irvine, United States of America

## Abstract

Platinum drug-resistance in ovarian cancers mediated by anti-apoptotic proteins such as Bcl-xL is a major factor contributing to the chemotherapeutic resistance of recurrent disease. Consequently, concurrent inhibition of Bcl-xL in combination with chemotherapy may improve treatment outcomes for patients. Here, we develop a mathematical model to investigate the potential of combination therapy with ABT-737, a small molecule inhibitor of Bcl-xL, and carboplatin, a platinum-based drug, on a simulated tumor xenograft. The model is calibrated against *in vivo* experimental data, wherein xenografts established in mice were treated with ABT-737 and/or carboplatin on a fixed periodic schedule. The validated model is used to predict the minimum drug load that will achieve a predetermined level of tumor growth inhibition, thereby maximizing the synergy between the two drugs. Our simulations suggest that the infusion-duration of each carboplatin dose is a critical parameter, with an 8-hour infusion of carboplatin given weekly combined with a daily bolus dose of ABT-737 predicted to minimize residual disease. The potential of combination therapy to prevent or delay the onset of carboplatin-resistance is also investigated. When resistance is acquired as a result of aberrant DNA-damage repair in cells treated with carboplatin, drug delivery schedules that induce tumor remission with even low doses of combination therapy can be identified. Intrinsic resistance due to pre-existing cohorts of resistant cells precludes tumor regression, but dosing strategies that extend disease-free survival periods can still be identified. These results highlight the potential of our model to accelerate the development of novel therapeutics such as BH3 mimetics.

## Introduction

Although ovarian cancer accounts for only 3% of cancers in women, it is the fifth most common cause of cancer death in women in the developed world [1]. Primary treatment for advanced ovarian cancer consists of cytoreductive surgery followed by adjuvant chemotherapy. The chemotherapeutic regimen typically combines a taxane such as paclitaxel with a platinum-based drug such as carboplatin, which causes cell death by inducing DNA damage. While patients initially respond well to therapy, most ultimately relapse, with recurrent disease being associated with progressive resistance to platinum-based therapy [2], [3]. Consequently, 5-year survival rates for women with advanced ovarian cancer are only 30–40% [4].

Several factors may contribute to platinum drug-resistance (for a comprehensive review, see [5], [6]). Here, we are concerned with the contribution of the anti-apoptotic protein Bcl-xL to drug-resistance. Bcl-xL belongs to the Bcl-2 family of intracellular proteins that regulates programmed cell death, or apoptosis [7]. Previous studies have revealed a significant correlation between Bcl-xL expression and carboplatin-resistance [8]–[10], and increased sensitivity to standard chemotherapeutic agents of ovarian cancer cell lines when Bcl-xL expression is inhibited [11]–[13]. Therefore, concomitant inhibition of Bcl-xL in combination with adjuvant chemotherapy may improve treatment outcomes for ovarian cancer patients.

In [13], Witham et al. assess the therapeutic potential of treating ovarian cancer that express Bcl-xL with carboplatin and ABT-737, a small-molecule inhibitor of Bcl-xL. Results from *in vitro* cell proliferation assays revealed synergistic inhibition of cell-growth and more rapid apoptosis when carboplatin was combined with ABT-737, than when it was administered as a single agent. Further, the times at which the two drugs are administered were also shown to be an important determinant of therapy efficacy, with an ABT-737 dose immediately following carboplatin found to yield the greatest extent of cell death. To understand better these experimental findings, we have previously developed a biochemically-motivated model for the growth of *in vitro* ovarian cancer [14], that was validated against available experimental data. A key prediction of our model is that the experimentally observed synergy between ABT-737 and carboplatin is due to the increased dependence of DNA-damaged cells intracellular Bcl-xL.

Here, we develop a mathematical model of ovarian cancer xenograft growth to test the efficacy of combining ABT-737 and carboplatin for the treatment of ovarian cancers growing *in vivo*. While models of cancer therapy involving platinum-based compounds [15], [16] or drugs targeting the Bcl-2 family [17], [18] have been proposed, to the best of our knowledge this is the first attempt to model the effect of a combination of these drugs on tumor growth. We carefully account for the pharmacodynamics of both drugs. Further, since the active processing of administered drugs by the body may have a significant effect on their efficacy and how they interact, we also need to incorporate the pharmacokinetics of carboplatin and ABT-737 in our model. The model is parametrized using experimental data reported in [13], wherein monoclonal ovarian tumor xenografts established in mice were treated with fixed doses of carboplatin and ABT-737 administered periodically and time-courses of tumor growth inhibition recorded. The validated model is then used to identify dosing strategies for the treatment of monoclonal tumors, which either lead to fastest times to minimal residual disease or minimize total drug load to achieve a predetermined level of tumor growth inhibition. The infusion time of carboplatin doses is predicted to be an important determinant of the long-term response of tumors to therapy, this testable prediction underscoring the practical significance of our results.

As mentioned earlier, a major cause of long-term treatment failure in ovarian cancer patients is the emergence of carboplatin-resistance. Given its synergistic action with carboplatin, ABT-737 co-therapy has the potential to prevent or delay treatment failure. We investigate this potential in the case when resistance to carboplatin is driven by genetic or epigenetic aberrations. Such aberrations arise in two different ways. In *acquired resistance*, genetic mutations emerge after the administration of chemotherapy, as a result of a failure in DNA repair in cells treated with carboplatin. Alternatively, in *intrinsic resistance*, a small population of resistant cells may already be present before the administration of chemotherapy. By distinguishing between these alternative scenarios of drug resistance, our model represents a useful tool with which to design individualized treatment protocols targeted against carboplatin-resistance.

## Results

**Figure 1:**
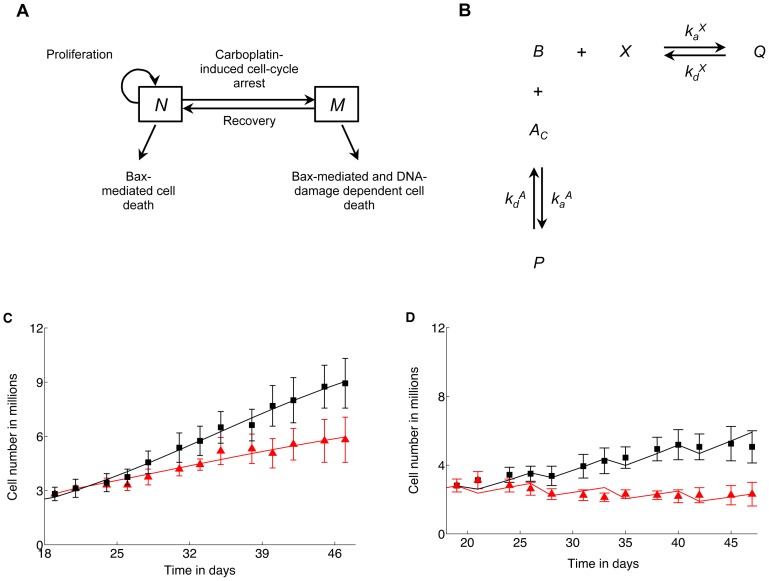
Model schematic and fits to experimental data. **A**, Model schematic. Ovarian cancer cells, 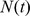 proliferate and undergo apoptosis at a rate dependent on intracellular Bax concentration. Administration of carboplatin induces DNA damage, leading to cell cycle arrest. Arrested cells, 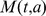 subsequently undergo apoptosis at a rate proportional to the amount of DNA damage sustained at the time of arrest and on their intracellular Bax concentration, and may also recover to the proliferating population. The rates at which 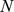 and 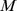 undergo cell death are elevated on application of ABT-737, which leads to increased levels of intracellular Bax. **B**, Intracellular reaction diagram of the heterodimerization reaction between Bcl-xL 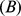 and Bax 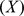 molecules, and the inhibition of Bcl-xL by intracellular ABT-737 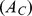. **C, D**, Fit to time-course tumor xenograft growth inhibition data taken from [13]. Briefly, ovarian cancer (IGROV-1 cell) xengrafts were established in mice and treatment started 19 days post-transplantation. Levels of tumor growth inhibition were recorded periodically. Experimental data is represented by black squares and red triangles, while solid curves show best fits. Values represent mean and standard deviation (from experimental data). **C**, Weekly treatment with vehicle (black squares and curve) or daily treatment with 100 mg/kg of ABT-737 (red triangles and curve). **D**, Weekly treatment with 30 mg/kg carboplatin (black squares and curve) or a combination of carboplatin and ABT-737 (red triangles and curve).

Our model of ovarian cancer xenograft growth and its response to carboplatin and ABT-737 therapy can be described in mathematical terms by a coupled system of ordinary and partial differential equations (further details are included in the Section S1 in File S1), which govern the temporal dynamics of the following key variables: the numbers of proliferating and arrested cancer cells; the concentrations of carboplatin and ABT-737 in circulatory and cancer tissue/intracellular compartments; and the intracellular concentrations of two members of the Bcl-family (Bcl-xL and Bax). A schematic detailing the response of the cancer cells to therapy is shown in Figure 1A, while Figure 1B depicts intracellular reactions between ABT-737 and Bcl-family proteins.

We calibrate our model by fitting time-course tumor xenograft growth inhibition data taken from [13], wherein ovarian cancer (IGROV-1 cell) xengrafts were established in mice and treatment in the form of 30 mg/kg carboplatin administered weekly, or 100 mg/kg ABT-737 administered daily, or a combination of both, started 19 days post-transplantation. The results of these fits are plotted in Figure 1. Following model calibration, a series of numerical experiments are carried out to identify dosing strategies that exploit the synergy between carboplatin and ABT-737. Since our model formulation is based on experiments that studied the response of monoclonal xenografts to therapy, we start by considering a tumor that comprises a homogenous population of carboplatin-sensitive cells. A major aim of our model is to investigate the potential of co-treatment with ABT-737 to prevent or delay the onset of carboplatin-resistance that is a leading cause of treatment failure. We therefore also simulate the treatment of a tumor that consists of carboplatin-sensitive and -resistant cells. The emergence of resistance under two distinct scenarios is considered: (i) acquired resistance resulting from faulty DNA damage repair; and (ii) intrinsic resistance resulting from a pre-existing population of resistant cells.

**Figure 2:**
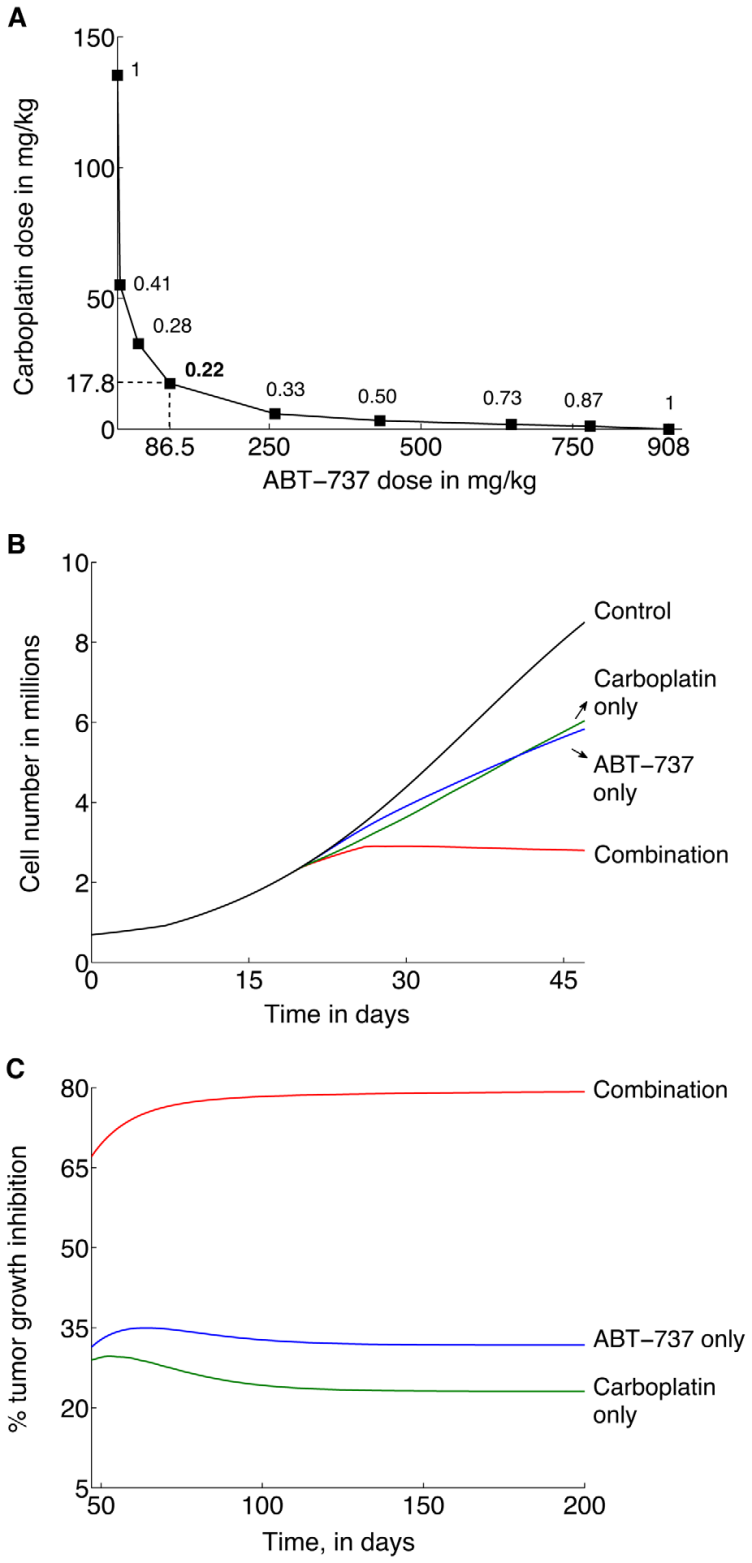
Optimizing carboplatin and ABT-737 doses when given in combination. **A**, Predicted Combination Index (CI) values computed for a desired tumor growth inhibition level of 67%, for various combinations of carboplatin and ABT-737. Following the experimental protocol in [13], treatment is initiated at 19 days post-transplantation and continued for 4 weeks. Simulations indicate that a combination of 17.8 mg/kg carboplatin given weekly combined with a daily dose of 86.5 mg/kg ABT-737 minimizes the CI and hence maximizes the synergy between the two drugs (dashed lines). **B**, Predicted tumor growth dynamics corresponding to the optimal dose combination found above. Plots show tumor cell numbers averaged over 7 days (the period of carboplatin administration) versus time. **C**, Predicted time-courses of tumor growth inhibition levels as compared to the control (no treatment) case, corresponding to the optimal dose combination found above.

**Figure 3:**
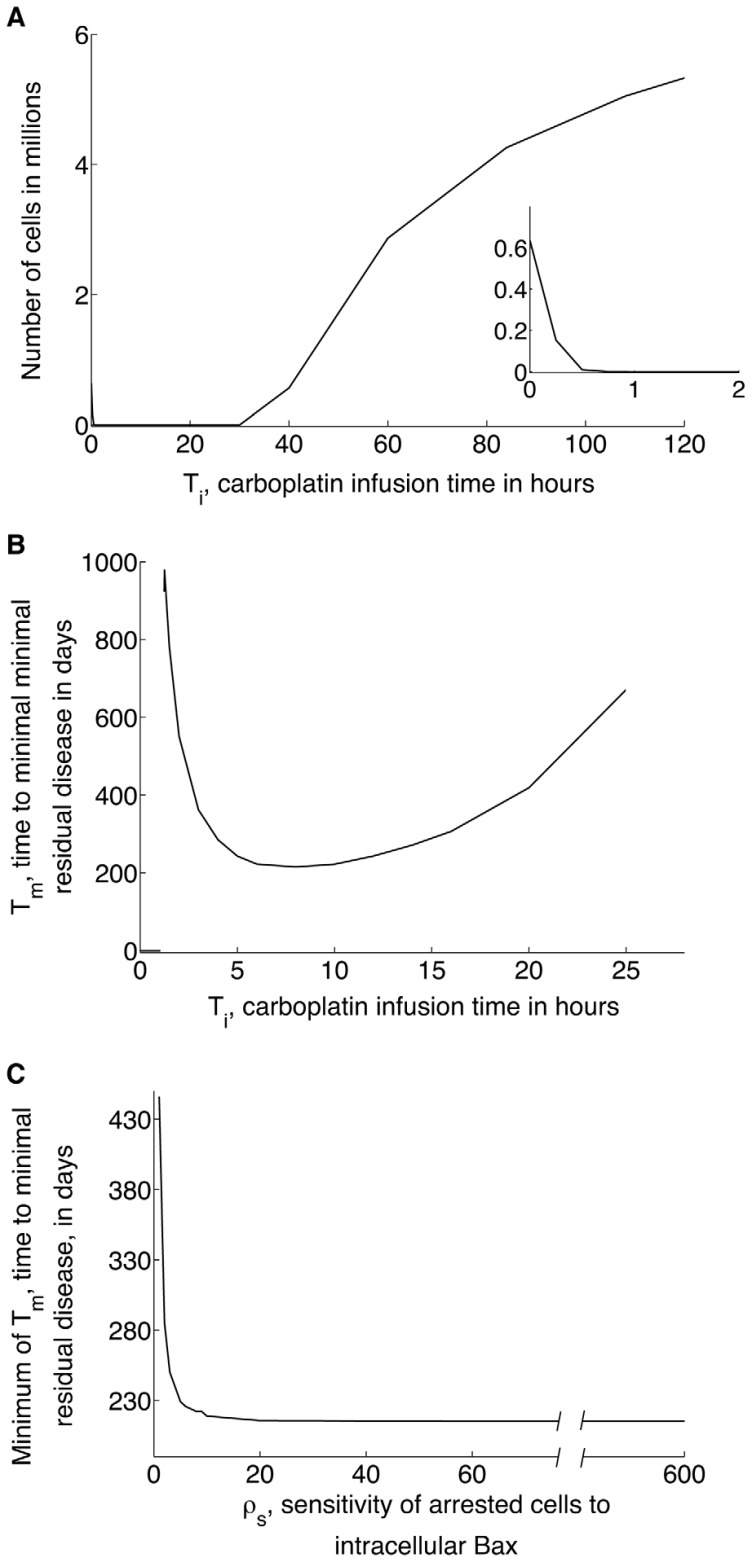
imulations showing long-term tumor xenograft response to combination therapy. Following the experimental protocol in [13], the administration of a weekly dose of 30 mg/kg carboplatin together with a daily dose of 100 mg/kg ABT-737 is simulated, and simulations allowed to run until tumor cell numbers averaged over 7 days (the period of carboplatin administration) achieve a steady-state. **A**, Predicted steady states of average tumor cell numbers as a function of carboplatin infusion time 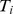. As 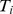 is increased from 0 hours (corresponding to a bolus dose) to 120 hours, the average tumor cell number steady-state decreases rapidly to 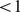 (see inset), and then increases, with minimal survival of tumor cells predicted for 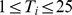. **B**, Predicted values of 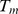, the length of time therapy must be administered to achieve minimal residual disease (defined as 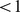 cell remaining) as 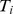 is varied between 1 and 25 hours. A minimum value of 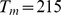 days is predicted for weekly carboplatin infusions lasting 8 hours. **C**, Minimum values of 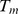 are predicted to decrease to 215 days as 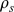, the arrested cell sensitivity to intracellular Bax is increased. In all cases, a carboplatin infusion time of 8 hours minimized the cure time.

**Figure 4:**
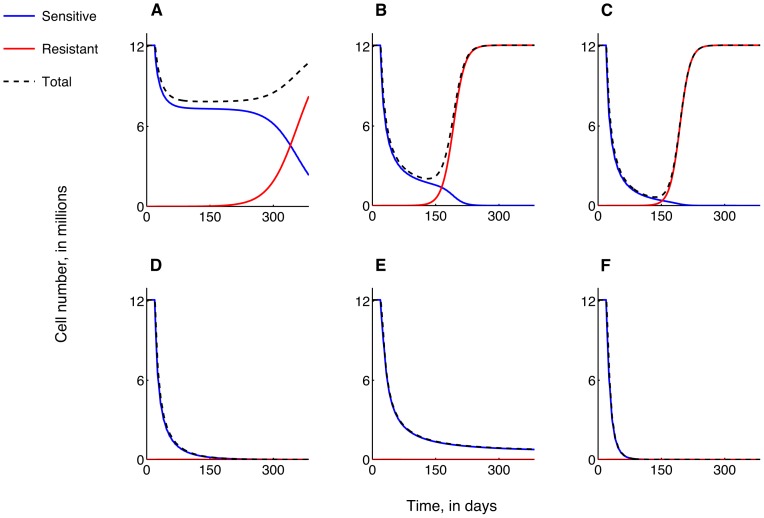
The emergence of acquired resistance to carboplatin therapy. DNA-mismatch repair in arrested cells recovering to the proliferating population is assumed to be the cause of resistance. The treatment of a late-stage tumor is simulated. Plots show 7 day-averages of carboplatin-sensitive (blue curve), carboplatin-resistant (red curve) and total (dashed black curve) tumor cell numbers versus time, as treatment strategy is varied. In all cases, therapy is administered for a period of 1 year. Increasing the weekly bolus dose of carboplatin administered as a single agent from **A**, 30 mg/kg, **B**, 300 mg/kg, to **C**, 800 mg/kg, cannot prevent the emergence of carboplatin-resistance. **D**, In fact, a weekly dose of 1300 mg/kg carboplatin is required to induce tumor regression. **E**, A combination of 30 mg/kg carboplatin delivered weekly as a bolus together with 100 mg/kg ABT-737 delivered daily is predicted to prevent the emergence of resistance and lead to tumor growth control at 6.5% of pre-treatment levels. **F**, The same combination dose, with carboplatin delivered via a 8-hour infusion is predicted to induce tumor regression within 150 days.

**Figure 5:**
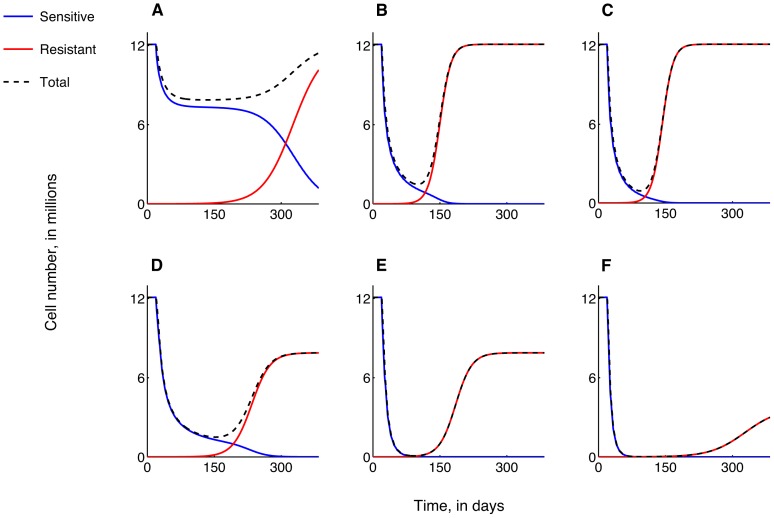
Intrinsic resistance to carboplatin therapy. A small fraction (1 in 60,000) of carboplatin-resistant cells is assumed to be present prior to treatment initiation. The treatment of a late-stage tumor is simulated. Plots show 7 day-averages of carboplatin-sensitive (blue curve), carboplatin-resistant (red curve) and total (dashed black curve) tumor cell numbers versus time, as treatment strategy is varied. In all cases, therapy is administered for a period of 1 year. Increasing the weekly bolus dose of carboplatin administered as a single agent from **A**, 30 mg/kg, **B**, 600 mg/kg, to **C**, 1300 mg/kg, cannot prevent the onset of carboplatin-resistance, with overall tumor size returning to pre-treatment levels eventually. **D, E, F**, In fact, a combination therapy of 30 mg/kg carboplatin delivered weekly as a bolus (**D**) or via 8-hour infusion (**E, F**) together ABT-737 delivered daily at a dose of 100 mg/kg (**D, E**) or 500 mg/kg (**F**) is also unable to prevent the emergence of resistance. However, combination therapy is predicted to result in partial tumor growth control, with a steady-state tumor size reaching 65.2% of pre-treatment levels for a combination of 30 mg/kg carboplatin and 100 mg/kg ABT-737 (**D, E**). Increasing ABT-737 dosage to 500 mg/kg is predicted to induce extended periods of disease-free survival, as evidenced by a dip in the total tumor size graph (**F**).

Following [13], in all simulations carboplatin is assumed to be administered on a weekly schedule and ABT-737 on a daily schedule. When simulating monoclonal xenograft treatment, the initial number of tumor cells is calculated from the size of tumors at the initiation of therapy. As can be seen from the cell number time-courses in Figure 1D, the weekly administration of carboplatin induces oscillations in tumor size. Therefore, cell numbers averaged over the period of carboplatin administration (7 days) are used to make quantitative comparisons between tumor responses to various treatment strategies (see Figures 2–5). Tumors are assumed to have achieved a steady-state average size if the relative change in average tumor cell numbers between successive weeks is less than 0.001% and time to minimal residual disease 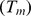 is defined as the period for which therapy must be administered so that the average number of cells is less than 1.

### Optimal Relative Doses for Monoclonal Tumors

We consider the following optimization problem in the treatment of cancers with a combination of two or more drugs – “What drug doses achieve a predetermined level of cell kill while minimizing patient drug load?”

For illustrative purposes, we use our model to predict the optimal doses of carboplatin and ABT-737 required to achieve a 67% growth inhibition in monoclonal tumors at the end of 4 weeks of therapy (that is, the treatment time reported in [13]). Note that minimizing the drug load is equivalent to minimizing the Combination Index 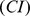 of the two drugs, defined as 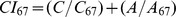 [19], subject to the constraint that 4 weeks post-therapy, tumor size is 33% of its untreated value. Here, 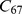 and 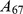 are the amounts of drugs required to achieve a 67% growth inhibition when administered as single-agents, 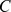 is the weekly bolus dose of carboplatin and 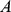 is the daily bolus dose of ABT-737 when these drugs are used in combination. 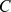 is varied between 0 and 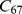, and the constraint used to generate values of 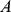. This represents an optimization problem for the function 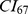 in a single variable 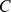 that assumes values on a closed and bounded interval and, hence, 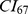 attains its minimum therein.

A phase diagram of the drug doses required to inhibit tumor growth by 67% after 4 weeks is shown in Figure 2A, with ABT-737 doses plotted along the abscissa and carboplatin doses plotted along the ordinate. 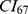 values for various dose combinations are enumerated along the curve. A weekly bolus of 17.8 mg/kg carboplatin combined with a daily bolus dose of 86.5 mg/kg ABT-737 is predicted to minimize the 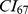 (dashed lines). We remark that similar curves can be computed for other levels of tumor growth inhibition at the end of a fixed time period, and for other treatment strategies. The tumor cell time-course data corresponding to these optimal doses presented in Figure 2B reveal that the levels of tumor growth inhibition after 4 weeks of treatment with either 86.5 mg/kg ABT-737 administered as a single agent or 17.8 mg/kg carboplatin administered weekly as a single agent are 31.4% and 28.9% respectively. The predicted level of growth inhibition when these doses are combined is 67%.

Simulating treatment for periods longer than 4 weeks with drug doses fixed at the above values further underscores the synergy between the two drugs. Figure 2C shows that if the tumor is allowed to reach its (average) steady state size, the levels of tumor growth inhibition decrease to 31.7% and 23.1% when ABT-737 or carboplatin are applied as monotherapy, respectively, whereas combination treatment results in a 79.2% tumor growth inhibition in the long term.

### Optimal Dose Scheduling for Monoclonal Tumors

We next use our model to identify an optimal dose-delivery strategy when monoclonal tumors are treated with a combination of ABT-737 and carboplatin for long times. Following the experimental protocol in [13], the doses of carboplatin and ABT-737 are fixed at 30 mg/kg and 100 mg/kg, respectively. In particular, the dependence of tumor response on two key parameters, the carboplatin infusion time 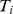, and the sensitivity 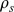 of arrested cells to changes in intracellular Bax, is investigated.

With 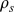 fixed at its baseline value 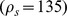, steady-state values of average cell number are calculated as 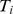 varies between 0 hours (bolus dose) and 120 hours. Figure 3A reveals that minimal survival of tumor cells is predicted for 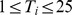. If 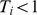 hour, even though significant levels of long-term tumor growth inhibition can be achieved (see inset), complete remission is not possible. Additionally, for 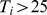 hours, the level of growth inhibition decreases rapidly as 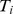 increases. In Figure 3B, we show how Tm, the length of time for which treatment must be administered in order to achieve minimal survival of tumor cells varies when 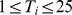. As Ti increases from 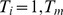 decreases, attaining a minimum of 215 days when carboplatin is administered via weekly infusions lasting 8 hours, and then increases for larger values of 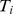.


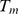
 depends not only on the carboplatin infusion time 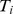, but also on 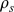, the sensitivity of arrested cells to changes in intracellular Bax. For each value of 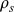 between 0 and 600, the minimum of 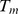 is calculated as 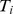 is varied between 0 and 120 hours. Figure 3C shows that this minimum decreases rapidly as 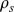 increases from 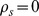, plateauing at a value of 215 days for large values of 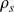. In all considered cases, 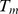 attains its minimum for 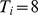 hours (data not shown).

### Emergence of Acquired Resistance

We now investigate the potential of ABT-737 co-therapy to prevent or delay the emergence of carboplatin-resistance. Since ovarian cancers are frequently diagnosed at a late stage [20], we simulate the application of therapy to tumors that have already attained their maximal untreated size (carrying capacity). As several carboplatin-sensitive and resistant ovarian cancer cell lines have been shown to have similar sensitivity to ABT-737 [13], carboplatin-sensitive and -resistant tumor cells are assumed to be equally sensitive to ABT-737 in our model.

We first assume that there is a small probability that cells recovering from a carboplatin-induced state of arrest experience DNA damage, resulting in a resistant phenotype. We assume further that no resistant cells exist at the start of treatment. Figure 4A–D shows predicted average cell number time-courses for a tumor treated with increasing weekly bolus doses of carboplatin only. A weekly dose of 1300 mg/kg is required to affect a cure and prevent the emergence of resistance (Figure 4D). Smaller doses result in transient decreases in tumor size, with the tumor eventually recovering to its untreated size (Figure 4A–C) due to the dominance of resistant cells. In contrast, Figure 4E reveals that a weekly bolus dose of 30 mg/kg carboplatin combined with a daily dose of 100 mg/kg ABT-737 may prevent the onset of carboplatin-resistance and result in tumor-growth control at 6.5% of its untreated level at steady-states. Significantly, the same combination, with carboplatin administered as an 8-hour infusion instead of as a bolus, is predicted to result in tumor remission within 150 days of treatment (Figure 4F).

### Optimal Dose Scheduling for Monoclonal Tumors

Finally, we consider the response of a tumor which contains a small population of carboplatin-resistant cells at the start of treatment. Figure 5A–C shows predicted average cell number time-courses when the tumor is treated with increasing weekly bolus doses of carboplatin alone. Now, even doses in excess of 1300 mg/kg (Figure 5C) are unable to induce sustained tumor regression. Further, in contrast to the case of acquired resistance, a weekly bolus dose of 30 mg/kg carboplatin combined with a daily dose of 100 mg/kg ABT-737 is unable to prevent the onset of carboplatin-resistance (Figure 5D), with the tumor recovering to 65.2% of its untreated size after a transient decline in cell numbers. Figure 5E shows that administering carboplatin as an 8-hour infusion results in the tumor reaching a much lower minimum at around day 150 of treatment, indicating a possible period of disease-free survival, defined as clinically undetectable disease. However, resistant cells eventually dominate and the tumor escapes from therapy-induced growth control. Increasing the daily ABT-737 dosage to 500 mg/kg in combination with 30 mg/kg carboplatin given as a 8-hour infusion is predicted to increase the disease-free survival time, and lead to a greater level of long-term growth control (Figure 5F).

## Discussion

The development of carboplatin-resistance is a major factor hampering the successful treatment of ovarian cancer with standard chemotherapy (carboplatin+paclitaxel). This resistance may be mediated in part by members of the Bcl-2 family that regulate cellular apoptosis. Consequently, it has recently been proposed [13] that inhibiting anti-apoptotic proteins such as Bcl-xL may improve the efficacy of carboplatin. A critical challenge in the development of such novel therapeutics is to arrive at treatment strategies that maximally exploit any synergy between the drugs, and to identify patients who might benefit from such a combination.

In this article, we have presented a mathematical model of the growth of an ovarian cancer xenograft and its treatment with carboplatin, and ABT-737, a small molecule inhibitor of Bcl-xL. The main goal of this research was to identify protocols for the treatment of ovarian cancer *in vivo* that exploit the molecular basis of synergy between the two drugs. To this end, we explicitly incorporated the intracellular regulation of apoptosis by the Bcl-2 family of proteins, and introduced a cell-age structured model to simulate the effect of carboplatin on cell fate. Further, to reflect the *in vivo* setting, detailed pharmacokinetics of carboplatin and ABT-737 were also included. The model was validated by fitting time-courses of ovarian cancer xenograft growth inhibition reported in [13]. The good agreement between model simulations and experimental data give confidence that the model is able accurately to describe the underlying biology.

We first simulated the treatment of tumors that consist of a monoclonal population of cells. Doses of carboplatin and ABT-737 required for a certain level of tumor growth inhibition were calculated, which minimize patient drug load and hence maximize the synergy between the two drugs. The method we describe here can in principle be applied to any drug combination and can easily be tailored to account for side effects of the drugs under evaluation, once appropriate data is available to support the model. Using our approach, physicians can estimate the impact of different doses of drugs in combination on the therapeutic response of patients. As combination therapies are increasingly being applied in oncology, models such as the one we present here can lead to significant time and cost savings by minimizing the choices that need to be examined experimentally. Further, such modeling can also be used to arrive at optimal individualized treatment strategies for evaluating combinations of chemotherapeutic drugs in clinical trials.

An equally important application of the model is predicting the long-term response to therapy. An exhaustive search of parameter space revealed a control parameter that is predicted to be a critical determinant of tumor growth dynamics when prolonged administration of carboplatin and ABT-737 co-therapy is simulated. This is the infusion time of the weekly dose of carboplatin. Carboplatin is typically administered as an infusion lasting 15 minutes or more when given in combination with paclitaxel. In fact, it has been proposed that the efficacy of carboplatin is directly linked to its AUC (Area Under the Curve) value, and the Calvert formula currently used to determine its clinical doses makes use of this fact [21]. However, our model simulations suggest that when combined with ABT-737, the efficacy of combination therapy is determined not only by the AUC of carboplatin, but also by the duration of drug infusion. Our simulations predict that weekly carboplatin infusions lasting between 1 and 25 hours combined with daily doses of ABT-737 result in the minimal survival of tumor cells. This is because, when administered as a rapid infusion, the peak tissue concentration of carboplatin is high, and a high level of DNA damage is induced in cancer cells from which they are unlikely to recover, irrespective of changes in intracellular Bcl-xL levels. Consequently, the synergy between the carboplatin and ABT-737 is muted. Equally, slower infusions of carboplatin result in extremely low tissue concentrations of the drug, so that the level of DNA damage induced in cells is minimal, and even with inhibited Bcl-xL levels, arrested cells are likely to recover to the proliferating pool. Note that since ABT-737 was simulated to be administered daily and has a slow clearance rate, its intracellular concentration rapidly achieves a non-zero mean (see Figure S2D). Therefore, changing the infusion time of ABT-737 administration is not predicted to alter our predictions.

Further, numerical simulations were carried out to identify the carboplatin infusion time that results in the fastest time to minimal residual disease. These times were observed to decrease rapidly to a minimum value as the sensitivity of arrested cells to intracellular Bax concentration 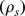 was increased. The parameter 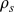 is a quantitative measure of the synergy between the carboplatin and ABT-737 and can be important in identifying a subclass of patients who would most benefit from such a combination of drugs. However, ?s cannot be directly measured from experiments. As demonstrated here, we can infer its value by fitting model simulations to tumor xenograft growth inhibition data when both drugs are given in combination.

ABT-737 co-therapy is now being developed to improve the efficacy of carboplatin, and may aid in delaying the onset of chemoresistance in ovarian cancers. We therefore investigated the therapeutic potential of combinations of ABT-737 and carboplatin to treat ovarian cancers in which carboplatin-resistance arises in two distinct scenarios. Genetic mutations leading to resistance may be acquired as a result of faulty DNA damage repair when cells try to recover from carboplatin administration. Carboplatin-resistance may also be an intrinsic property of the cancer, stemming from resistant cells present when treatment starts. A key strength of our approach is the ability to distinguish between these scenarios. For instance, in the case of acquired resistance, model simulations predicted that preventing cells that have undergone carboplatin-induced DNA-damage from recovering and returning to the proliferating population precludes the emergence of resistance. However, the amount of carboplatin required to achieve this as a single-agent may be toxic for the host and thus not feasible. In contrast, combination therapy at low doses, with carboplatin administered optimally as described earlier, is sufficient to prevent the onset of resistance. When resistance to carboplatin is intrinsic, tumor remission is no longer feasible, but our model can be applied to identify dosing strategies that extend periods of disease-free survival. It has been proposed that the development of chemoresistance may result from insufficient exposure of tumor cells to drugs [22], and our simulations further accentuate the dangers of under-treatment.

The model presented in this article has the potential to accelerate the translation from bench-to-bedside of novel therapeutics such as ABT-737, and to reduce the costs associated with drug development. However, the eventual clinical application of our model will require the validation of its predictions with further experiments. For instance, tumor xenograft growth inhibition experiments with varying doses of carboplatin and ABT-737 alone, and in combination would be extremely helpful in fine-tuning the functional responses of cancer cells to therapy. Measuring the relative constitutive expression levels of the Bcl-2 family would improve the accuracy of the quantitative description of the ABT-737-targeted intracellular apoptosis pathway. Detailed pharmacokinetic studies of ABT-737, which include the temporal dynamics of its intracellular concentration, would help in a better parameterization of our model. Finally, experimentally validating our model predictions relating to the optimal time of infusion of carboplatin when co-administered with ABT-737 could constitute a significant breakthrough in the treatment of ovarian cancers, and solid tumors in general.

A limitation of our approach is that while we have incorporated carboplatin-resistance by simulating a completely resistant cell line, in practice a human tumor may contains many different populations of cells with varying levels of resistance to carboplatin, and sensitivities to ABT-737. Further, resistance to therapy can arise from multiple mechanisms. Consequently, in future versions of our model we will incorporate a greater diversity of cellular phenotypes. We also plan to include the administration of paclitaxel as a third drug in the combination chemotherapy of ovarian cancers, and replace ABT-737 with ABT-263, its orally available analogue currently under phase I clinical trials for the treatment of a number of solid tumors [23], [24].

Finally, the work presented here illustrates how mathematical modeling has the potential to support the preclinical and clinical development of novel cancer therapeutics. There are numerous parameters that affect the outcome of drug evaluation, and it may not be feasible to address all of these experimentally. Quantitative modeling represents a powerful resource to optimize the likelihood of the successful development of targeted therapies.

## Materials and Methods

### Model Foundation

our model of ovarian cancer xenograft growth and treatment consists of a coupled system of ordinary and partial differential equations (full model equations are listed in section S1 in File S1), which govern the temporal dynamics of the following key variables: N(t) and M(t,a), the numbers of proliferating and arrested cancer cells (in millions) respectively; 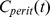, 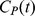 and 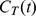, the concentrations in 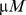 of intraperitoneal, plasma and tissue carboplatin respectively; 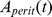, 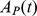 and 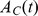, the concentrations in nM of intraperitoneal, plasma and intracellular ABT-737 respectively; and 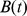, 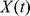, 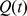 and 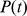, the intracellular concentrations in nM of Bcl-xL, Bax, Bcl-xLBax complex and Bcl-xLABT-737 complex respectively. Here, time t is measured in days, and a is a time-like variable, representing the period of time a cell has spent in a growth-arrested state. A schematic detailing the response of the cancer cells to therapy is shown in Figure 1A. In the sections that follow, the principles underlying our model formulation are introduced.

### Dynamics of Proliferating Cells

Equation A models the growth of proliferating ovarian cancer cells, 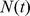.
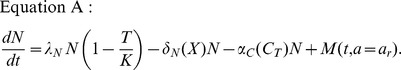



The growth rate of untreated tumor xenografts is typically exponential at early times, and plateaus as they become larger [25]. Consequently, we assume that ovarian cancer cells grow logistically in the absence of treatment, with growth rate 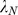 and carrying capacity 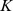. We remark that models of periodic chemotherapy based on the logistic equation have been proposed previously [26]–[29]. Arrested cells 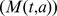 are assumed to compete for space with proliferating cells so that 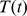 is the total (proliferating+arrested) number of cancer cells at time 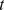. The parameters 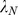 and 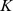 are chosen by fitting time-courses of cell numbers to data from untreated tumor xenografts in [13], as shown in Figure 1C (black curve).

We account for the regulation of cell death by the Bcl-2 family of proteins in the following way. For simplicity, and in the absence of appropriate experimental data, we represent each of the pro- and anti-apoptotic sub-families of the Bcl-2 family by single variables. Given the specificity of ABT-737 for Bcl-2 and Bcl-xL, and its similar binding affinity for both these molecules [30], we represent the anti-apoptotic members of the Bcl-2 family by Bcl-xL. Further, Bax is taken to represent the pro-apoptotic proteins since it is the members of the Bax-like subfamily which controls the release of cytochrome c from the mitochondria that leads to caspase activation, that is followed by cell death [7]. The rate of cell death 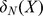 is consequently assumed to be an increasing function of free intracellular Bax 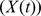.

Pharmacologic therapy is applied periodically in the form of ABT-737 or carboplatin, alone or in combination. ABT-737 increases the rate of cell death 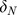, while carboplatin induces DNA damage and subsequent cell arrest at a rate 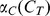, which depends on tissue carboplatin concentration 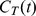. The final term in Equation A represents the rate at which arrested cells recover and return to the proliferating pool.

We now describe the effect on the growing tumor of the application of carboplatin and ABT-737.

#### Effect of ABT-737 on Proliferating Cells

Upon application, ABT-737 enters the proliferating tumor cells where it binds to, and occupies Bcl-xL (see Figure 1B). This results in a build-up of free Bax that was previously sequestered in the form of Bcl-xLBax heterodimers, thereby increasing the rate of cell death. Parameters relating to the death rate 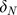 of proliferating cells are chosen by fitting time-courses of estimated cell numbers to tumor xenograft growth inhibition data taken from [13], wherein IGROV-1 xenografts established in mice were treated daily with a fixed dose of ABT-737 administered intraperitoneally for 4 weeks. The best fit is shown in Figure 1C (red curve). The reaction diagram in Figure 1B representing ABT-737 pharmacodynamics is translated into a system of ordinary differential equations using the principle of mass balance.

#### Effect of Carboplatin on Proliferating Cells

The cytotoxicity of carboplatin is primarily due to damage induced by the formation of intra- and interstrand adducts at the nucleophilic N7 sites in the DNA. This damage stimulates the activation of downstream pathways that lead to cell cycle arrest, followed by either survival if the DNA damage is repairable, or apoptosis [6]. Consequently, upon drug application, proliferating cells are assumed to undergo cell cycle arrest at a rate 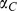 that is assumed to be an increasing and saturating function of the tissue carboplatin concentration, 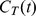, so that as the drug dose (and correspondingly the level of DNA damage) increases, the rate of cell cycle arrest also increases up to a maximum level.

### An Age-structured Model of Arrested Cell Dynamics

The arrested cells are removed to a separate compartment, where they either undergo apoptosis or recover and return to the proliferating population (a schematic is shown in Figure 1A). In [13], the initiation of apoptosis was routinely observed in cells 12–16 hours post carboplatin administration. Consequently, arrested cell dynamics are described by the following partial differential equation derived by applying the general McKendrick equation that is widely used to model age-structured populations [31].
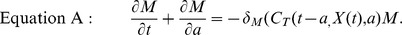



Here the rate of arrested cell death 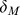 is taken to be a function of the time a for which the cells have been arrested. Cell cytotoxicity has been found to correlate linearly with the amount of platinum bound to the DNA, and hence the extent of DNA damage [6]. Accordingly, the rate of arrested cell death is taken to be linearly proportional to 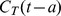, the amount of tissue carboplatin at the time of cell-cycle arrest. Next, as in the case of proliferating cells, when ABT-737 is co-administered with carboplatin, it is taken up by the arrested cells where it binds to, and occupies Bcl-xL (see Figure 1B), causing a build-up of free Bax, 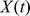. Further, in [14] the observed synergy between carboplatin and ABT-737 was shown to be due to an increased dependence of DNA-damaged cells on Bcl-xL for survival. Accordingly, the rate of arrested cell death 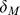 is taken also to be proportional (with constant of proportionality 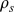) to 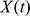. 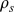 is an important parameter in our model: it represents the sensitivity of the arrested cells to changes in Bax, and hence provides a quantitative measure of the degree of synergy between carboplatin and ABT-737. Equation B is solved subject to the boundary condition:
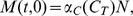
which represents the rate of cell arrest (see Equation A). Finally, the length of time for which a cell can remain in an arrested state is limited. We therefore assume that cells that have not undergone apoptosis after a characteristic time 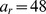 hours recover and return to the proliferating population, this return being instantaneous so that 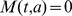 for 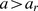. Additionally, the number of arrested cells at time 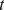 is given by 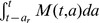.

Parameters relating to the rate at which proliferating cells become growth arrested 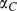 and the rate at which arrested cells die 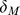 are chosen by fitting time-courses of estimated numbers of cells to tumor xenograft growth inhibition data taken from [13], wherein IGROV-1 xenografts established in mice were treated weekly with a fixed dose of carboplatin administered intravenously for 4 weeks as a single agent, or in combination with a fixed daily dose of ABT-737. The best fits are shown in Figure 1D.

The functional forms used in our model for the response of proliferating and arrested cells to carboplatin and ABT-737 also account for their pharmacokinetics, which are described below.

### Carboplatin Pharmacokinetics

Carboplatin was administered intraperitoneally on a weekly schedule in [13] and its pharmacokinetics are assumed to be governed by the following 3-comparment model. Experimental evidence suggests that small molecular weight drugs (molecular weight of carboplatin = 371.2 Da [32]) delivered intraperitoneally are readily absorbed through the peritoneal vasculature to enter systemic circulation [33], [34]. Consequently, the peritoneal cavity is taken to be the first compartment. Since the time-activity curve of carboplatin in the blood plasma of mice has been shown to be biphasic [32], the circulatory system together with highly vascularized and well-perfused organs are taken to be the second or central compartment, and the peripheral organs and tissues with relatively poor vascular perfusion account for the third compartment. As tumor vasculature is characterized by its poor functional quality, and is highly disorganized [35], we assume that the tumor resides in the peripheral pharmacokinetic compartment of carboplatin. Details regarding carboplatin pharmacokinetics and pharmacodynamics are provided in section S3 in File S1 and Figure S2. Related parameter values are provided in Table S3.

We investigate the effect on tumor response of administering the same dose of carboplatin as either a rapid infusion or bolus, or a continuous infusion lasting several hours, when given in combination with ABT-737. In fact, the time of infusion 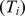 of each dose of carboplatin is predicted to be a critical parameter in determining the therapeutic efficacy of combination therapy.

### ABT-737 Pharmacokinetics

ABT-737 was administered intraperitoneally on a daily schedule in [13] and its pharmacokinetics are assumed to be governed by the following 3-comparment model. Since ABT-737 is a low molecular weight drug (molecular weight = 813.4 Da [24]), as in the case of carboplatin, the peritoneal cavity is taken as the first compartment, and the systemic circulation as the central (and second) compartment. In our model, we explicitly account for the regulation of cell death by the Bcl-2 family of proteins. Therefore, a third intracellular compartment, into which the drug permeates from the systemic circulation, is included. Details regarding ABT-737 pharmacokinetics and the intracellular regulation of cell death are provided in section S2 in File S1 and Figure S1. Related parameter values are provided in Tables S1 and S2.

We remark that given that the circulation half-life of ABT-737 is several hours (see File S1), administering it daily ensures that carboplatin-arrested cells are exposed to it, irrespective of the time of cell arrest.

#### The emergence of carboplatin-resistance

When considering the emergence of resistance to carboplatin, the proliferating cell population is subdivided into two classes - carboplatin-sensitive and carboplatin-resistant. Following [13] where ovarian cancer cell lines with different sensitivities to carboplatin were observed to be comparably responsive to ABT-737, both carboplatin-sensitive and resistant cells are assumed to be equally sensitive to ABT-737.

### Model Parametrization and Simulation

Details of parameter estimation described in this section, and a list of parameter values can be found in section S5 in File S1 and Table S4. All model simulations are carried out in Matlab, a technical computing language, the numerical methods being described in Section S4 of the supplemental File S1. Figure S3 shows typical model simulations. A sensitivity analysis on the estimated parameter values can be found in Figure S4 and section S5 of the supplemental File S1.

## Supporting Information

Figure S1 **ABT-737 pharmacokinetics in a mouse.** **A**, ABT-737 is periodically administered intraperitoneally (i.p.), into the peritoneal cavity from where it enters the systemic circulation. From here, ABT-737 enters the intracellular compartment, and is also cleared from the body. Figures showing **B**, intraperitoneal, **C**, plasma and **D**, intracellular ABT-737 concentration time-courses when 100 mg/kg of the drug is administered daily starting on day 5. **E**, Resultant intracellular Bcl-xL (black curve) and Bax (red curve) concentration time-courses.
(TIF)

Figure S2 **Carboplatin pharmacokinetics in a mouse.** **A**, Carboplatin is periodically administered intraperitoneally (i.p.), into the peritoneal cavity from where it enters the systemic circulation. From here, carboplatin is distributed to peripheral organs and tissues with poor vascular perfusion, and is also cleared from the body. Figures showing plasma (black curve) and peripheral tissue (red curve) carboplatin concentration time-courses corresponding to a dose of 30 mg/kg, given **B**, as a bolus, or **C**, as a continuous infusion lasting 12 hours.
(TIF)

Figure S3 **Tumor xenograft response to 30 mg/kg of carboplatin-only therapy.** Carboplatin administration as a bolus dose every 7 days, starting on day 19 (black arrow) is simulated. Figure shows total cell number (red curve) and total cell number averaged over the period of therapy administration (black curve) versus time.
(TIF)

Figure S4 **Parameter sensitivity analysis.** **A–E**, Model sensitivity to key parameters. Variation of the parameters from their baseline values is plotted on the x-axis. The % change in the Euclidean norm of the error over its value from performing fits of the model to experimental data (see Figures 1B,C in main manuscript) is plotted on the y-axis. **F**, Predicted average total (black curve), proliferating (red curve) and growth arrested (blue curve) tumor cell numbers at the end of 4 weeks of treatment of a tumor xenograft with 30 mg/kg carboplatin administered weekly, as the time of infusion of each dose is varied.
(TIF)

File S1 **Supplementary Information. Section S1: Model Equations. Section S2: ABT-737 Pharmacokinetics and the Intracellular Regulation of Cell Death. Section S3: Carboplatin Pharmacokinetics. Section S4: Simulation Methodology. Section S5: Parameter Estimation for Monoclonal Tumor Xenograft Growth Treatment.**
(PDF)

Table S1 **List of parameter values relating to ABT-737 pharmacokinetics and pharmacodynamics.**
(PDF)

Table S2 **List of non-dimensional parameter values relating to ABT-737 pharmacokinetics and pharmacodynamics.**
(PDF)

Table S3 **List of parameter values relating to carboplatin pharmacokinetics.**
(PDF)

Table S4 **List of parameter values relating to xenograft growth and treatment.**
(PDF)
