# Supplementary figures and images for: Exploiting the Synergy between Carboplatin and ABT-737 in the Treatment of Ovarian Carcinomas
Source: PLoS One. 2014 Jan 6;9(1):e81582. doi: 10.1371/journal.pone.0081582 (PMC3882219; doi:10.1371/journal.pone.0081582)

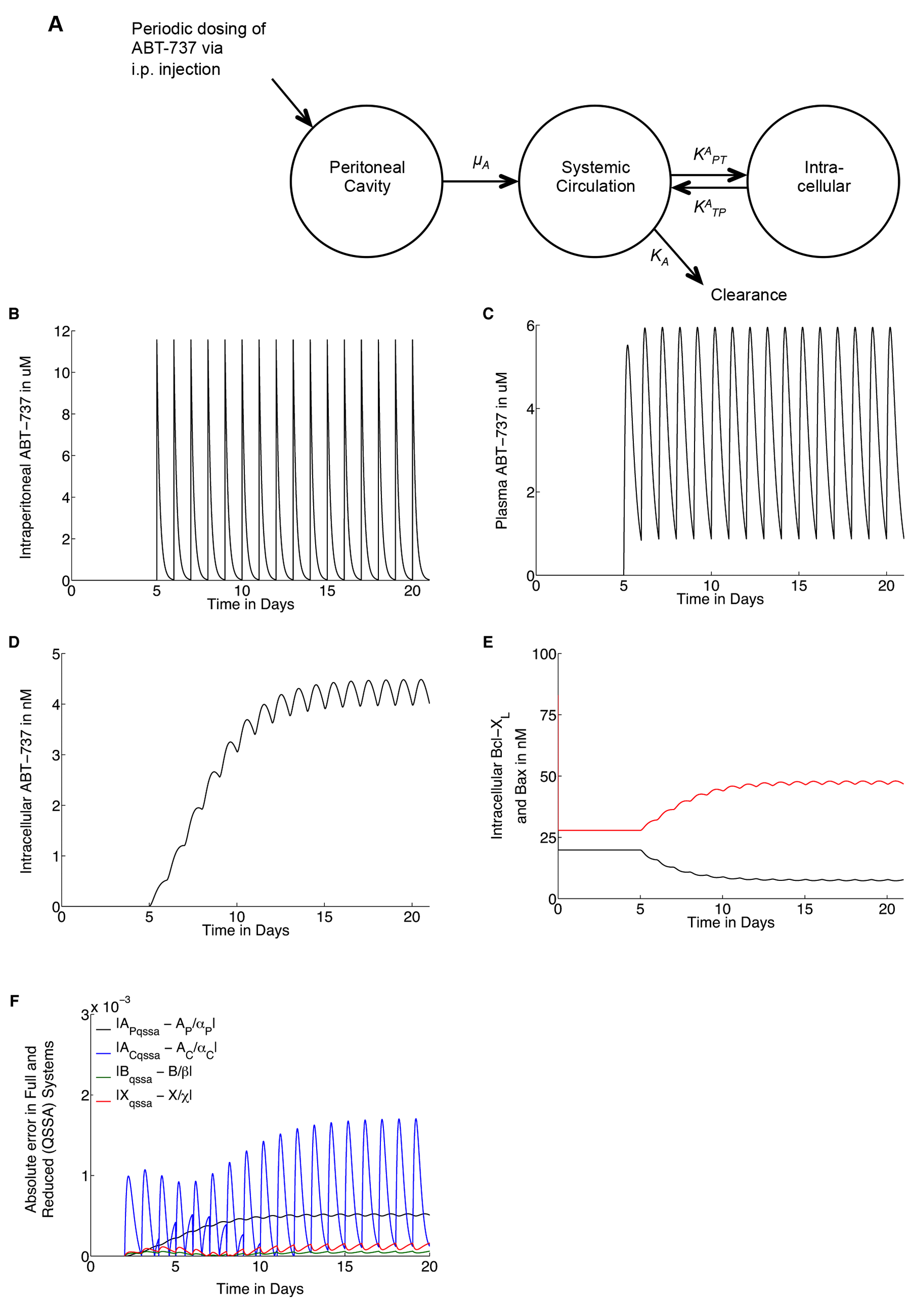

Supplement: Figure S1 — ABT-737 pharmacokinetics in a mouse. A, ABT-737 is periodically administered intraperitoneally (i.p.), into the peritoneal cavity from where it enters the systemic circulation. From here, ABT-737 enters the intracellular compartment, and is also cleared from the body. Figures showing B, intraperitoneal, C, plasma and D, intracellular ABT-737 concentration time-courses when 100 mg/kg of the drug is administered daily starting on day 5. E, Resultant intracellular Bcl-xL (black curve) and Bax (red curve) concentration time-courses. (TIF) [file pone.0081582.s001.tif]

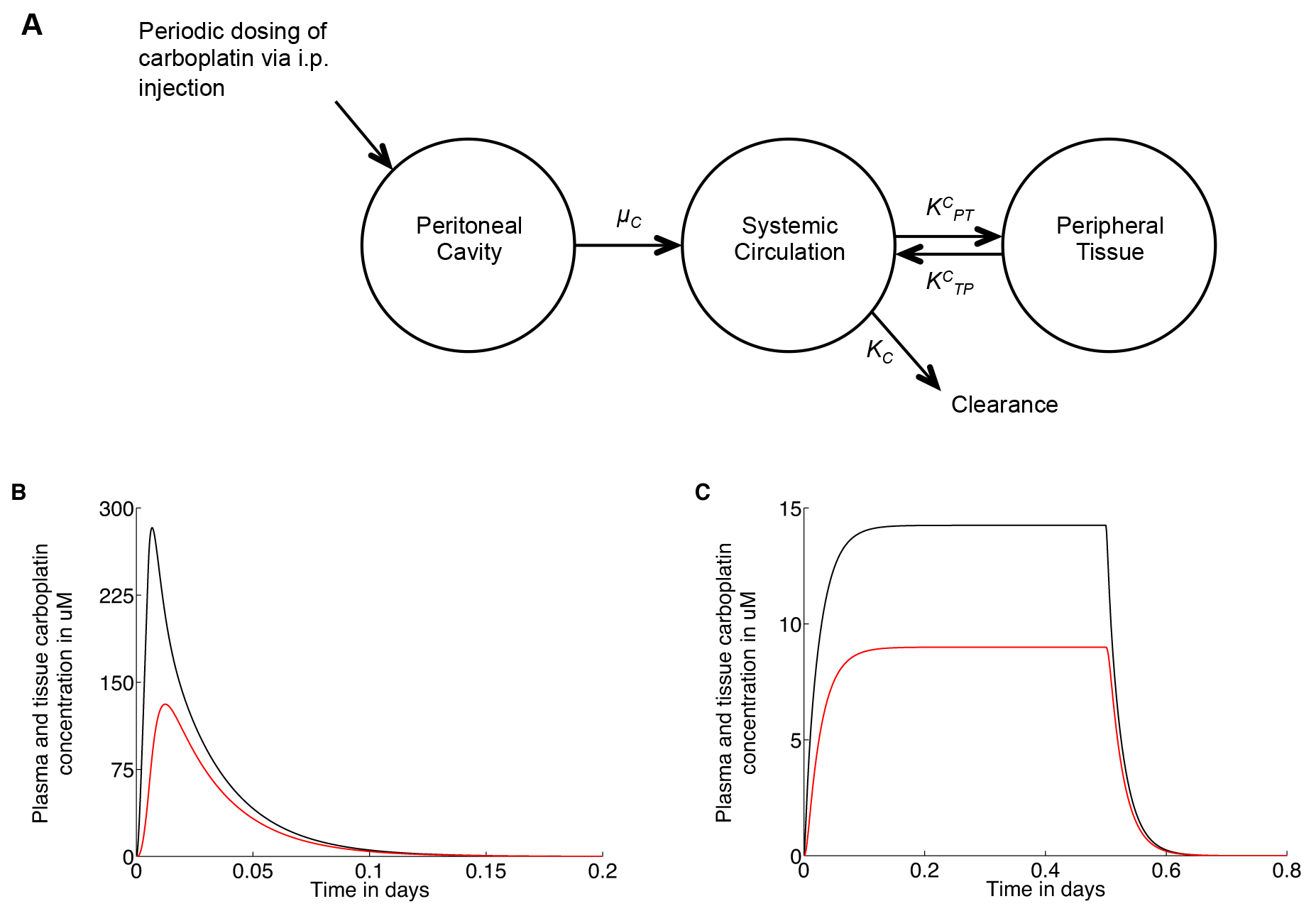

Supplement: Figure S2 — Carboplatin pharmacokinetics in a mouse. A, Carboplatin is periodically administered intraperitoneally (i.p.), into the peritoneal cavity from where it enters the systemic circulation. From here, carboplatin is distributed to peripheral organs and tissues with poor vascular perfusion, and is also cleared from the body. Figures showing plasma (black curve) and peripheral tissue (red curve) carboplatin concentration time-courses corresponding to a dose of 30 mg/kg, given B, as a bolus, or C, as a continuous infusion lasting 12 hours. (TIF) [file pone.0081582.s002.tif]

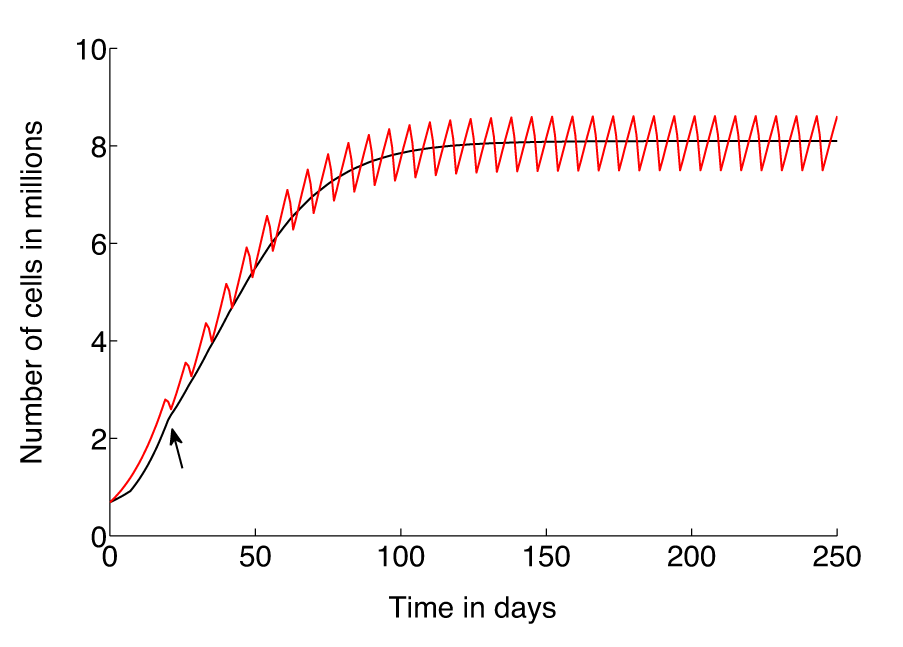

Supplement: Figure S3 — Tumor xenograft response to 30 mg/kg of carboplatin-only therapy. Carboplatin administration as a bolus dose every 7 days, starting on day 19 (black arrow) is simulated. Figure shows total cell number (red curve) and total cell number averaged over the period of therapy administration (black curve) versus time. (TIF) [file pone.0081582.s003.tif]

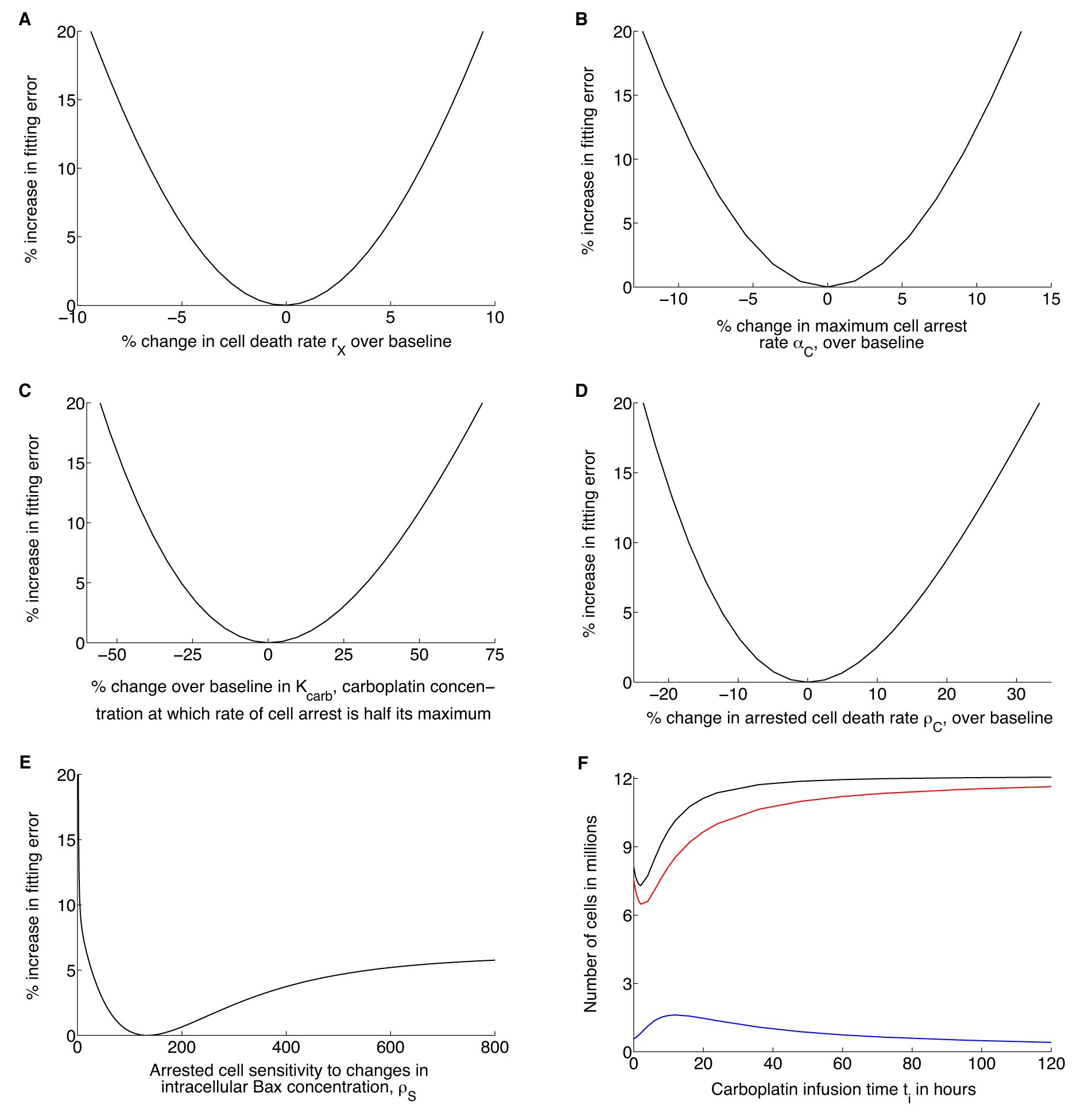

Supplement: Figure S4 — Parameter sensitivity analysis. A–E, Model sensitivity to key parameters. Variation of the parameters from their baseline values is plotted on the x-axis. The % change in the Euclidean norm of the error over its value from performing fits of the model to experimental data (see Figures 1B,C in main manuscript) is plotted on the y-axis. F, Predicted average total (black curve), proliferating (red curve) and growth arrested (blue curve) tumor cell numbers at the end of 4 weeks of treatment of a tumor xenograft with 30 mg/kg carboplatin administered weekly, as the time of infusion of each dose is varied. (TIF) [file pone.0081582.s004.tif]
