# Supplementary material for: Exploiting the Synergy between Carboplatin and ABT-737 in the Treatment of Ovarian Carcinomas
Source: PLoS One. 2014 Jan 6;9(1):e81582. doi: 10.1371/journal.pone.0081582 (PMC3882219; doi:10.1371/journal.pone.0081582)
Supplement: File S1 — Supplementary Information. Section S1: Model Equations. Section S2: ABT-737 Pharmacokinetics and the Intracellular Regulation of Cell Death. Section S3: Carboplatin Pharmacokinetics. Section S4: Simulation Methodology. Section S5: Parameter Estimation for Monoclonal Tumor Xenograft Growth Treatment. (PDF) [file pone.0081582.s005.pdf]

Supplementary Information

S1 Model Equations

The full system of equations used to model the treatment with carboplatin and ABT-737 of an ovarian cancer xenograft is presented below, together with notation used.

Notation

t	Time (days)
a	Time (days)
$N(t)$	Number of carboplatin-sensitive proliferating ovarian cancer cells (millions)
$M(t,a)$	Number of arrested cancer cells that have spent time a in the arrested state (millions/day)
$C_{perit}(t)$	Intraperitoneal carboplatin concentration (μM)
$C_P(t)$	Plasma carboplatin concentration (μM)
$C_T(t)$	Tissue carboplatin concentration (μM)
$A_{perit}(t)$	Intraperitoneal ABT-737 concentration (nM)
$A_P(t)$	Plasma ABT-737 concentration (nM)
$A_C(t)$	Intracellular ABT-737 concentration (nM)
$B(t)$	Intracellular Bcl-X_L concentration (nM)
$X(t)$	Intracellular Bax concentration (nM)
$P(t)$	Intracellular Bcl-X_L-ABT-737 complex concentration (nM)
$Q(t)$	Intracellular Bcl-X_L-Bax complex concentration (nM)

Proliferating Cell Equation

$$\frac{dN}{dt} = \underbrace{\lambda_N \, N \left(1 - \frac{N + \int_{t-a_r}^{t} M(t,a)da}{K}\right)}_{\text{logistic growth}} - \underbrace{\delta_N(X)\, N}_{\substack{\text{Bcl-X}_\text{L}\text{-media-}\\ \text{ted cell death}}} - \underbrace{\alpha_C(C_T)\, N}_{\substack{\text{carboplatin-ind-}\\ \text{uced cell arrest}}} + \underbrace{M(t, a = a_r)}_{\substack{\text{recovery to}\\ \text{proliferating pool}}}, \quad \text{(S1.1)}$$

with initial condition $N(t=0) = 0.69$ million cells.

Arrested Cell Equation

$$\frac{\partial M}{\partial t} + \frac{\partial M}{\partial a} = -\underbrace{\delta_M(C_T(t-a), X(t), a)\, M}_{\substack{\text{Bcl-X}_\text{L}\text{- and carboplatin-} \\ \text{mediated arrested cell death}}}, \tag{S1.2}$$

with boundary condition

$$M(t,0) = \alpha_C(C_T)\, N, \tag{S1.3}$$

and with initial condition $M(t=0, a) = 0$ million cells per day.

Carboplatin Pharmacokinetics

$$\frac{dC_{perit}}{dt} = \underbrace{-\mu_C\, C_{perit}}_{\substack{\text{extravasation to} \\ \text{systemic circulation}}} + \underbrace{K_0(T_i)\, H(T_i - t)}_{\substack{\text{dosage with time} \\ \text{of infusion } T_i}}, \tag{S1.4}$$

$$\frac{dC_P}{dt} = \underbrace{\mu_C \frac{V_{perit}^C}{V_P^C} C_{perit}}_{\substack{\text{source from} \\ \text{peritoneal cavity}}} - \underbrace{K_C\, C_P}_{\text{clearance}} - \underbrace{K_{PT}^C\, C_P}_{\substack{\text{extravasation to} \\ \text{peripheral tissue}}} + \underbrace{K_{TP}^C \frac{V_T^C}{V_P^C} C_T}_{\substack{\text{source from} \\ \text{peripheral tissue}}}, \tag{S1.5}$$

$$\frac{dC_T}{dt} = \underbrace{K_{PT}^C \frac{V_P^C}{V_T^C} C_P}_{\substack{\text{source from} \\ \text{systemic circulation}}} - \underbrace{K_{TP}^C\, C_T}_{\substack{\text{extravasation to} \\ \text{systemic circulation}}}, \tag{S1.6}$$

with initial conditions $C_{perit}(t=0) = C_P(t=0) = C_T(t=0) = 0$ μM.

ABT-737 Pharmacokinetics and Intracellular Signal Transduction

$$\frac{dA_{perit}}{dt} = \underbrace{-\mu_A\, A_{perit}}_{\substack{\text{extravasation to} \\ \text{systemic circulation}}}, \tag{S1.7}$$

$$\frac{dA_P}{dt} = \underbrace{\mu_A \frac{V^A_{perit}}{V^A_P} A_{perit}}_{\substack{\text{source from}\\ \text{peritoneal cavity}}} - \underbrace{K_A A_P}_{\text{clearance}} - \underbrace{K^A_{PC} A_P}_{\substack{\text{extravasation to intra-}\\ \text{cellular compartment}}} + \underbrace{K^A_{CP} \frac{V^A_C}{V^A_P} A_C}_{\substack{\text{source from intra-}\\ \text{cellular compartment}}}, \tag{S1.8}$$

$$\frac{dA_C}{dt} = \underbrace{-k^A_a A_C B + k^A_d P}_{\text{reaction with Bcl-X}_\text{L}} + \underbrace{K^A_{PC} \frac{V^A_P}{V^A_C} A_P}_{\substack{\text{source from}\\ \text{systemic circulation}}} - \underbrace{K^A_{CP} A_C}_{\substack{\text{extravasation to}\\ \text{systemic circulation}}}, \tag{S1.9}$$

$$\frac{dB}{dt} = \underbrace{-k^A_a A_C B + k^A_d P}_{\text{reaction with ABT-737}} - \underbrace{k^X_a X B + k^X_d Q}_{\text{reaction with Bax}}, \tag{S1.10}$$

$$\frac{dX}{dt} = \underbrace{-k^X_a X B + k^X_d Q}_{\text{reaction with Bcl-X}_\text{L}}, \tag{S1.11}$$

$$\frac{dP}{dt} = \underbrace{k^A_a A_C B - k^A_d P}_{\text{ABT-737-Bcl-X}_\text{L}\text{ reaction}}, \tag{S1.12}$$

$$\frac{dQ}{dt} = \underbrace{k^X_a X B - k^X_d Q}_{\text{Bax-Bcl-X}_\text{L}\text{ reaction}}, \tag{S1.13}$$

with initial conditions $A_{perit}(t=0) = A_P(t=0) = A_C(t=0) = 0$ nM; $B(t=0) = 75$ nM; $X(t=0) = 83.33$ nM; and $P(t=0) = Q(t=0) = 0$ nM.

In the above equations, the Bax-dependent death rate of proliferating cells is

$$\delta_N(X) = r_X X; \tag{S1.14}$$

the rate of proliferating cell arrest induced by carboplatin is

$$\alpha_C(C_T) = r_C \frac{C_T^2}{C_T^2 + K^2_{carb}}; \tag{S1.15}$$

the carboplatin- and Bax-dependent rate of arrested cell death is

$$\delta_M(C_T(t-a), X(t), a) = (\rho_C + \rho_S r_X (X - X_{phys}))\, C_T(t-a)\, H(a - a_{char}); \tag{S1.16}$$

the carboplatin infusion dosage function is

$$K_0(T_i) = \begin{cases} 0, & \text{if } T_i = 0 \\ D_C / \left(V^C_{perit} w_C T_i\right), & \text{if } T_i > 0 \end{cases}; \tag{S1.17}$$

and the Heaviside function is defined as

$$H(x) = \begin{cases} 0, & \text{if } x \leq 0 \\ 1, & \text{if } x > 0 \end{cases}. \tag{S1.18}$$

In the sections that follow, we explain how the model equations were developed, discuss parameter estimates and outline the simulation methodology used to generate the numerical results.

S1.1 Modeling resistance to carboplatin

Carboplatin-resistant cells are denoted with the variable $R(t)$. They are assumed to be insensitive to treatment with carboplatin, but as sensitive to ABT-737 as N cells. In our model, the following two cases are considered: (i) acquired resistance where cells recovering from a carboplatin-induced state of arrest experience aberrant DNA damage repair, resulting in a resistant phenotype, and (ii) intrinsic resistance where a small population of resistant cells is present initially. The equations governing the evolution of N and R cell dynamics for the two cases of interest are presented below.

Acquired resistance

$$\frac{dN}{dt} = \lambda_N N \left(1 - \frac{T}{K}\right) - \delta_N(X) N - \alpha_C(C_T) N + p M(t, a = a_r), \tag{S1.19}$$

$$\frac{dR}{dt} = \lambda_R R \left(1 - \frac{T}{K}\right) - \delta_R(X) R + (1 - p) M(t, a = a_r), \tag{S1.20}$$

subject to initial conditions $N(t = 0) = 12.08$ million cells and $R(t = 0) = 0$ million cells, and where $T(t) = N(t) + R(t) + \int_{t-a_r}^{t} M(t, a) da$ is the total number of cells at time t and p is the probability of aberrant DNA damage repair.

Intrinsic resistance

$$\frac{dN}{dt} = \lambda_N N \left(1 - \frac{T}{K}\right) - \delta_N(X)\, N - \alpha_C(C_T)\, N + M(t, a = a_r), \tag{S1.21}$$

$$\frac{dR}{dt} = \lambda_R R \left(1 - \frac{T}{K}\right) - \delta_R(X)\, R, \tag{S1.22}$$

subject to initial conditions $N(t = 0) = 12.078$ million cells and $R(t = 0) = 0.002$ million cells, and where, as before, $T(t) = N(t) + R(t) + \int_{t-a_r}^{t} M(t, a)da$ is the total number of cells at time t.

In equations (S1.20) and (S1.22), the Bax-dependent death rate of resistant cell death is taken as

$$\delta_R(X) = r_X\, X. \tag{S1.23}$$

S2 ABT-737 Pharmacokinetics and the Intracellular Regulation of Cell Death

In this section we explain how we model ABT-737 pharmacokinetics and pharmacodynamics.

S2.1 Model Derivation

In experiments described in [1], ABT-737 (an 813 Da compound) is administered intraperitoneally via periodic bolus injections. The following 3-compartment model is assumed to govern its pharmacokinetics and a schematic is shown in Figure S1A. The first compartment is the peritoneal cavity into which the drug is injected. Experimental evidence suggests that small molecular weight drugs are readily absorbed through the peritoneal vasculature from here they enter the systemic circulation [2, 3], which is taken to be the second compartment. From here the drug may be cleared from the body, or enter the third, intracellular compartment where it interacts with the Bcl-2 family of proteins (specifically, Bax and

Bcl-X_L), as represented by the following reactions:

$$A_C \; + \; B \; \underset{k_d^A}{\overset{k_a^A}{\rightleftharpoons}} \; P, \qquad\qquad X \; + \; B \; \underset{k_d^X}{\overset{k_a^X}{\rightleftharpoons}} \; Q.$$

Here, k_a^A and k_a^X are the association rate constants for Bcl-X_L binding ABT-737 and Bax respectively, and k_d^A and k_d^X are the corresponding dissociation rate constants. By appealing to the principal of mass balance, these reactions may be translated into a system of time-dependent differential equations describing how the concentrations of intracellular ABT-737, Bax, Bcl-X_L, ABT-737-Bcl-X_L complex and Bax-Bcl-X_L vary with time. It is straightforward to show that the complete system of equations governing the pharmacokinetics and intracellular dynamics of ABT-737 is given by:

$$\frac{dA_{perit}}{dt} = -\mu_A\, A_{perit}, \tag{S2.1}$$

$$\frac{dA_P}{dt} = \mu_A\, \frac{V_{perit}^A}{V_P^A}\, A_{perit} - K_A\, A_P - K_{PC}^A\, A_P + K_{CP}^A\, \frac{V_C^A}{V_P^A}\, A_C, \tag{S2.2}$$

$$\frac{dA_C}{dt} = -\,k_a^A\, A_C\, B + k_d^A\, P + K_{PC}^A\, \frac{V_P^A}{V_C^A}\, A_P - K_{CP}^A\, A_C, \tag{S2.3}$$

$$\frac{dB}{dt} = -\,k_a^A\, A_C\, B + k_d^A\, P - k_a^X\, X\, B + k_d^X\, Q, \tag{S2.4}$$

$$\frac{dP}{dt} = k_a^A\, A_C\, B - k_d^A\, P, \tag{S2.5}$$

$$\frac{dX}{dt} = -\,k_a^X\, X\, B + k_d^X\, Q, \tag{S2.6}$$

$$\frac{dQ}{dt} = k_a^X\, X\, B - k_d^X\, Q. \tag{S2.7}$$

Here, $A_{perit}(t)$, $A_P(t)$ and $A_C(t)$ represent the concentrations of ABT-737 in the peritoneal, circulatory and intracellular compartments, respectively; V_{perit}^A, V_P^A and V_C^A are the assumed constant volumes of

distribution of ABT-737 in these compartments; and μ_A is the rate of extravasation of ABT-737 from the peritoneal cavity into the systemic circulation, K_A is the rate of its clearance from circulation; and K_{PC}^A and K_{CP}^A are the rates of entry of ABT-737 from the systemic circulation into the intracellular compartment and vice versa, respectively. In the absence of additional information, we assume $V_{perit}^A \approx V_P^A \approx V_C^A$. Equation (S2.1) can be solved explicitly to give $A_{perit}(t) = \alpha_{perit}\, e^{-\mu_A t}$, where $\alpha_{perit} = A_{perit}(t=0)$. When simulating the periodic administration of the drug (with period $\tau_A \geq 1$ day), the expression for the intraperitoneal concentration of ABT-737 is modified to $A_{perit}(t) = \alpha_{perit}\, e^{-\mu_A\, rem(t,\tau_A)}$, where $rem(x, y)$ denotes the remainder when x is divided by y. We remark that equations (S2.4)-(S2.7) imply the following conservation laws for intracellular Bcl-X_L and Bax:

$$B(t) + P(t) + Q(t) \;=\; \beta \qquad \text{and} \qquad X(t) + Q(t) \;=\; \chi, \tag{S2.8}$$

where β and χ are positive constants representing the total amounts in a given cell of Bcl-X_L and Bax, respectively.

S2.2 Parameter Estimation

A list of parameter values with sources is provided in Table S1. For clarity, we discuss the estimation of those parameters values which could not be determined directly from the literature. Since we require that ABT-737 is cleared from the peritoneal cavity within 24 hours (see previous subsection), we estimate that μ_A, its rate of extravasation from the peritoneal cavity into the systemic circulation, satisfies $\mu_A \geq 5.30$ per day. Here we take $\mu_A = 6$ per day. The rate K_{CP}^A of ABT-737 leakage from the intracellular compartment to the circulation, and its rate K_{PC}^A of entry from the circulation to the intracellular compartment are chosen to ensure that the intracellular concentration of ABT-737 is comparable to typical expression levels of the Bcl-2 family of proteins (estimated to be of the order of a few nM [4, 5]).

Equations (S2.1)-(S2.7) are simulated using the parameter values in Table S1 to generate typical ABT-737, Bcl-X_L and Bax time-courses, for a therapy schedule in which 100 mg/kg ABT-737 is given daily, the first treatment time at $t = 5$ days. The results presented in Figure S1 reveal that ABT-737 is rapidly

cleared from the peritoneal cavity after each dose (Figure S1B). Correspondingly, ABT-737 levels in the systemic circulation settle to periodic oscillations, with a maximum of 5.9 μM and a minimum of 0.9 μM (see Figure S2C). The intracellular ABT-737 concentration is also periodic with a maximum of 4.5 nM and a minimum of 4.0 nM (see Figure S2D). Prior to therapy, the intracellular concentrations of Bcl-X_L and Bax are at their steady state values of 19.83 and 27.83 nM, respectively (see Figure S2E). When therapy is administered, the Bcl-X_L concentration falls rapidly, eventually oscillating between values of 7.3 and 7.8 nM, while the Bax concentration rises and undergoes periodic solutions with levels ranging from 46.7 and 48.0 nM. The period of the oscillations in the intracellular levels of ABT-737, Bcl-X_L and Bax correspond to the period of drug administration (1 day), with ABT-737 and Bax oscillating in phase, and Bcl-X_L oscillating π radians out of phase. This phase shift is a result of the inhibitory action of ABT-737 on Bcl-X_L.

S2.3 Asymptotic Analysis

The parameter estimates in Table S1 reveal that equations (S2.1)-(S2.7) operate on multiple different time scales. We now conduct an asymptotic analysis of these equations in order to understand the system dynamics on the longer time-scale that corresponds to therapy application and tumor growth. The system of equations is first recast in dimensionless variables, which are denoted with an asterisk. The following rescaling is chosen: $A^*_{perit} = A_{perit}/\alpha_{perit}$, $A^*_P = A_P/\alpha_P$ where $\alpha_P = \max_{t\in[0,\infty)} A_P(t)$, $A^*_C = A_C/\alpha_C$ where $\alpha_C = \max_{t\in[0,\infty)} A_C(t)$, $B^* = B/\beta$, $P^* = P/\beta$, $Q^* = Q/\beta$ and $X^* = X/\chi$. Finally, time is scaled with the timescale associated with consecutive applications of therapy so that $t^* = t/\tau_A$. Introducing these variables in equations (S2.1)-(S2.7) (and omitting asterisks for ease of notation), we obtain the following non-dimensional system.

$$A_{perit}(t) = e^{-\bar{\mu}_A rem(t,1)}, \tag{S2.9}$$

$$\frac{dA_P}{dt} = \bar{\eta}_A \, A_{perit} - \bar{K}_A \, A_P - \epsilon \, \bar{\kappa}_0 \, (\bar{\kappa}_1 \, A_P - \bar{\kappa}_2 \, A_C) \, , \tag{S2.10}$$

$$\epsilon \frac{dA_C}{dt} \;=\; -\bar{\gamma}_1\,(\bar{\nu}_1\,A_C\,B\,-\,\bar{\nu}_2\,P)\,+\,\epsilon\,(\bar{\kappa}_1\,A_P\,-\,\bar{\kappa}_2\,A_C)\,, \tag{S2.11}$$

$$\epsilon\,\frac{dB}{dt} \;=\; -\,(\bar{\nu}_1\,A_C\,B\,-\,\bar{\nu}_2\,P)\,-\,(\bar{\nu}_3\,X\,B\,-\,Q)\,, \tag{S2.12}$$

$$\epsilon\,\frac{dX}{dt} \;=\; -\bar{\gamma}_2\,(\bar{\nu}_3\,X\,B\,-\,Q)\,, \tag{S2.13}$$

where $P = 1 - B - Q$ and $Q = (1 - X)/\bar{\gamma}_2$ from the conservation laws stated in (S2.8).

We have introduced a small parameter $\epsilon = 1/(k_d^X\,\tau_A)$, which measures the ratio of the frequency of ABT-737 application, and the dissociation rate of the Bcl-X_L-Bax complex into its constituent species. The non-dimensional parameters (denoted by an overbar in the above equations) are defined in Table S2.

We approximate solutions of the model variables which are regular power series expansions in ϵ so that, for example,

$$A_P(t) \;=\; A_{P,0}(t)\,+\,\epsilon\,A_{P,1}(t)\,+\,\epsilon^2\,A_{P,2}(t)\,+\,\cdots$$

Substituting the above into equation (S2.10) gives:

$$\begin{aligned}\frac{d}{dt}\,(A_{P,0}+\epsilon A_{P,1}) \;=\;& \bar{\eta}_A\,A_{perit}\,-\,\bar{K}_A\,(A_{P,0}+\epsilon A_{P,1})\,-\,\epsilon\,\bar{\kappa}_0\,\bar{\kappa}_1\,(A_{P,0}+\epsilon A_{P,1})\\ &+\epsilon\,\bar{\kappa}_0\,\bar{\kappa}_2\,(A_{C,0}+\epsilon A_{C,1})\,+\,O(\epsilon^2).\end{aligned} \tag{S2.14}$$

Equating coefficients of $O(\epsilon^0)$ and $O(\epsilon^1)$ we get:

$$\frac{dA_{P,0}}{dt} \;=\; \bar{\eta}_A\,A_{perit}\,-\,\bar{K}_A\,A_{P,0}, \tag{S2.15}$$

$$\frac{dA_{P,1}}{dt} \;=\; -\bar{K}_A\,A_{P,1}\,-\,\bar{\kappa}_0\,\bar{\kappa}_1\,A_{P,0}\,+\,\bar{\kappa}_0\,\bar{\kappa}_2\,A_{C,0}. \tag{S2.16}$$

The same process applied to equations (S2.11)-(S2.13) gives:

$$0 = -\bar{\nu}_1 A_{C,0} B_0 + \bar{\nu}_2 P_0, \tag{S2.17}$$

$$0 = -\bar{\nu}_3 X_0 B_0 + Q_0, \tag{S2.18}$$

$$\frac{dA_{C,0}}{dt} = -\bar{\gamma}_1 \left(\bar{\nu}_1 A_{C,0} B_1 + \bar{\nu}_1 A_{C,1} B_0 - \bar{\nu}_2 P_1\right) + \left(\bar{\kappa}_1 A_{P,0} - \bar{\kappa}_2 A_{C,0}\right), \tag{S2.19}$$

$$\frac{dB_0}{dt} = -\left(\bar{\nu}_1 A_{C,0} B_1 + \bar{\nu}_1 A_{C,1} B_0 - \bar{\nu}_2 P_1\right) - \left(\bar{\nu}_3 X_0 B_1 + \bar{\nu}_1 X_1 B_0 - Q_1\right), \tag{S2.20}$$

$$\frac{dX_0}{dt} = -\bar{\gamma}_2 \left(\bar{\nu}_3 X_0 B_1 + \bar{\nu}_1 X_1 B_0 - Q_1\right), \tag{S2.21}$$

where $P_0 = 1 - B_0 - Q_0$ and $Q_0 = (1 - X_0)/\bar{\gamma}_2$. Equations (S2.17) and (S2.18) may be solved simultaneously to obtain the following expressions for B_0 and X_0 in terms of $A_{C,0}$:

$$B_0(A_{C,0}) = \frac{-\left(\zeta_1 A_{C,0} + \zeta_2\right) + \zeta_1 \sqrt{A_{C,0}^2 + \rho_1 A_{C,0} + \rho_2}}{\bar{\nu}_1 A_{C,0} + \bar{\nu}_2}, \tag{S2.22}$$

$$X_0(A_{C,0}) = -\left(\sigma_1 A_{C,0} + \sigma_2\right) + \sigma_1 \sqrt{A_{C,0}^2 + \rho_1 A_{C,0} + \rho_2}. \tag{S2.23}$$

The new parameters $\zeta_i, \sigma_i, \rho_i$ $(i = 1, 2)$ are defined in Table S2. Further, from equations (S2.19)- (S2.21), we deduce

$$\frac{dA_{C,0}}{dt} = \bar{\gamma}_1 \left(\frac{dB_0}{dt} - \frac{1}{\bar{\gamma}_2}\frac{dX_0}{dt}\right) + \left(\bar{\kappa}_1 A_{P,0} - \bar{\kappa}_2 A_{C,0}\right). \tag{S2.24}$$

Finally, a simplified differential equation for $A_{C,0}$ is obtained by substituting equations (S2.22) and (S2.23) into equation (S2.24). Finally, by neglecting terms of $O(\epsilon)$, we obtain the following approximation (in non-dimensional terms) to equations (S2.1)-(S2.7) which model ABT-737 pharmacokinetics and intra-

cellular dynamics, on the timescale of tumor growth.

$$A_{perit}(t) \quad = \quad e^{-\bar{\mu}_A \, rem(t,1)}, \tag{S2.25}$$

$$\frac{dA_P}{dt} \quad = \quad \bar{\eta}_A \, A_{perit} \, - \, \bar{K}_A \, A_P, \tag{S2.26}$$

$$B(A_C) \quad = \quad \frac{-\,(\zeta_1 \, A_C \, + \, \zeta_2) \, + \, \zeta_1 \, \sqrt{A_C^2 \, + \, \rho_1 \, A_C \, + \, \rho_2}}{\bar{\nu}_1 \, A_C \, + \, \bar{\nu}_2}, \tag{S2.27}$$

$$X(A_C) \quad = \quad -\,(\sigma_1 \, A_C \, + \, \sigma_2) \, + \, \sigma_1 \, \sqrt{A_C^2 \, + \, \rho_1 \, A_C \, + \, \rho_2}, \tag{S2.28}$$

$$\frac{dA_C}{dt} \quad = \quad (\bar{\kappa}_2 \, A_P \, - \, \bar{\kappa}_3 \, A_C) \, \frac{\bar{\gamma}_2}{\bar{\gamma}_2 \, - \, \bar{\gamma}_1 \, \bar{\gamma}_2 \, (dB/dA_C) \, + \, \bar{\gamma}_1 \, (dX/dA_C)}. \tag{S2.29}$$

We remark that numerical validation of the above analysis is provided by the excellent agreement between the solutions of the original system of equations (S2.1)-(S2.7) and those of the reduced system (S2.25)-(S2.29) (see Figure S1F).

S3 Carboplatin Pharmacokinetics

In this section, we explain how we model carboplatin pharmacokinetics.

S3.1 Model Derivation

In experiments described in [1], carboplatin is administered as a series of periodic intraperitoneal injections (or bolus doses). The following 3-compartment model is assumed to govern its pharmacokinetics and a schematic is shown in Figure S2A. As for ABT-737, the peritoneal cavity is the first compartment of interest. From here the drug extravasates into the systemic circulation, the second compartment. Experimental evidence suggests a biphasic plasma concentration-time curve for carboplatin [6]. Consequently, a third compartment, comprising tissues and organs with poor vascular perfusion, is also

included. Combining these effects, we deduce that he equations governing carboplatin pharmacokinetics are

$$\frac{dC_{perit}}{dt} = -\mu_C\, C_{perit} + K_0(T_i)\, H(T_i - t), \tag{S3.1}$$

$$\frac{dC_P}{dt} = \mu_C\, \frac{V^C_{perit}}{V^C_P}\, C_{perit} - K_C\, C_P - K^C_{PT}\, C_P + K_{TP}\, \frac{V^C_T}{V^C_P}\, C_T, \tag{S3.2}$$

$$\frac{dC_T}{dt} = K^C_{PT}\, \frac{V^C_P}{V^C_T}\, C_P - K^C_{TP}\, C_T. \tag{S3.3}$$

Here, $C_{perit}(t)$, $C_P(t)$ and $C_T(t)$ are the concentrations of carboplatin in the peritoneal, circulatory and intracellular compartments, respectively; V^C_{perit}, V^C_P and V^C_T are the assumed constant volumes of distribution of carboplatin in these compartments; μ_C is the rate of extravasation of carboplatin from the peritoneal cavity into the systemic circulation; K_C is the rate of its clearance from circulation; and K^C_{PT} and K^C_{TP} represent the rates of entry of carboplatin from the systemic circulation into the intracellular compartment and vice versa. We consider several delivery strategies for carboplatin, including bolus and continuous infusions. The parameter T_i measures the infusion time of carboplatin with $T_i = 0$ corresponding to a bolus dose. The function $K_0(T_i)$ is the rate of infusion, defined as:

$$K_0(T_i) = \begin{cases} 0, & \text{if } T_i = 0, \\ R_i / \left(V^C_{perit} w_C\right), & \text{if } T_i > 0, \end{cases} \tag{S3.4}$$

where R_i is the rate (in μg per day) at which carboplatin is being administered and w_C is its molecular weight. When carboplatin is administered as a series of periodic boluses, with period of therapy τ_C days, equation (S3.1) can be solved explicitly to give (cf. Equation (S2.9)):

$$C_{perit}(t) = C_{perit}(t=0)\, e^{-\mu_C\, rem(t,\tau_C)}, \tag{S3.5}$$

where $C_{perit}(t = 0)$ is the concentration of carboplatin in the intraperitoneal cavity immediately following an injection. When carboplatin is administered periodically as a continuous infusion, with each infusion

lasting T_i days and the period of therapy τ_C days, equation (S3.1) can be solved explicitly to give:

$$C_{perit}(t) \quad = \quad \begin{cases} \dfrac{K_0}{\mu_C}\left(1 - e^{-\mu_C \, rem(t,\tau_C)}\right), & \text{if } rem(t,\tau_C) \leq T_i \\ \dfrac{K_0}{\mu_C}\left(e^{\mu_C \, rem(T_i,\tau_C)} - 1\right) e^{-\mu_C \, rem(t,\tau_C)}, & \text{if } rem(t,\tau_C) > T_i \end{cases} . \tag{S3.6}$$

In the above discussion, $T_i < \tau_C$. Given its rapid rate of clearance from mouse plasma [6], we have also assumed that the intraperitoneal concentration of carboplatin at the end of each cycle of therapy is ~ 0. Thus, the complete system of equations governing the pharmacokinetics of carboplatin is given by equations (S3.2)- (S3.6).

We remark that if the amount of carboplatin to be administered in each round of therapy is fixed at D_C μg, the initial concentration of carboplatin in the intraperitoneal cavity immediately following a bolus dose is $C_{perit}(t=0) = \dfrac{D_C}{V^C_{perit} w_C}$ μM, while in the case of continuous infusion, the rates of infusion are given by $K_0 = \dfrac{D_C}{V^C_{perit} w_C T_i}$ μM per day and $R_i = \dfrac{D_C}{T_i}$ μg per day.

S3.2 Parameter Estimation

A list of parameter values with sources is provided in Table S3. For clarity, we discuss the estimation of those parameters values which could not be determined directly from the literature. The volume of distribution of carboplatin in the peritoneal cavity V^C_{perit} is taken to be the average of its volumes of distribution in the systemic circulation and peripheral tissue compartments. The rate of carboplatin extravasation from the peritoneal cavity into systemic circulation (μ_C) is taken to be the average of the rates of entry of carboplatin from the systemic circulation into the intracellular compartment and vice versa (K^C_{PT} and K^C_{TP} respectively).

In Witham et al. [1], a bolus dose of 30 mg/kg (equivalent to a dose $D_C = 600$ μg assuming a typical mouse weighs 200 g) was administered weekly to mice: we simulate equations (S3.2)- (S3.5) to generate similar plasma and tissue carboplatin concentration time-courses, using the equivalent dose $D_C = 600$ μg. The results plotted in Figure S2B reveal that plasma (black curve) and tissue (red curve) carboplatin

is cleared within 2-3 hours, and while the maximum plasma concentration $C^b_{Pmax} = 283.0\ \mu$M, the maximum tissue concentration is $C^b_{Tmax} = 131.1\ \mu$M. To illustrate the altered dynamics of carboplatin when it is administered via continuous infusion, we also simulate equations (S3.2) (S3.4) and (S3.6) for the same total dose D_C, and for an infusion time $T_i = 12$ hours. The results plotted in Figure S2C reveal that plasma (black curve) and tissue (red curve) carboplatin reach steady states of 14.3 μM and 9.0 μM, respectively within 3 hours of therapy application and are rapidly cleared at the end of the infusion.

S4 Simulation Methodology

The system of time-delayed ordinary, partial differential and algebraic equations used to simulate the treatment with carboplatin and ABT-737 of a growing tumor xenograft (equations (S1.1)-(S1.3), (S1.14)-(S1.18), (S2.25)-(S2.29) and (S3.2)-(S3.6)) is solved as follows. Due to their stiff nature, the drug pharmacokinetic and pharmacodynamic equations (S2.25)-(S2.29) and (S3.2)-(S3.6) are solved in the computing software Matlab using 'ode23s', an in-built solver for stiff equations. The following solution to the hyperbolic equation governing the evolution of arrested cells (S1.2) is constructed using the method of characteristics [7]:

$$M(t,a) \;=\; \alpha_C(C_T(t-a))\,N(t-a)\,e^{-C_T(t-a)\int_{t-(a-a_{char})}^{t}(\rho_C+\rho_S r_X(X(u)-X_{phys}))du}, \quad t \geq a \tag{S4.1}$$

The above equation and the solutions to the drug equations inform equation (S1.1), which governs the dynamics of proliferating cells. The fourth order explicit Runge-Kutta method is used to solve this equation.

Typical simulation results are presented in Figure S3. They show the response of a tumor xenograft to weekly boluses of 30 mg/kg carboplatin treatment starting on day 19, as in [1]. The periodic therapy induces oscillations in tumor size, and hence cell numbers. Averaging cell numbers over the period of carboplatin administration (black curve in Figure S3) facilitates quantitative comparisons between tumor responses to the various treatment strategies that are discussed in the manuscript.

Details of the code used for simulating our model are available upon request. Please direct queries to the corresponding author.

S5 Parameter Estimation for Monoclonal Tumor Xenograft Growth Treatment

Where possible, the values of parameters relating to the growth rate of the tumor xenograft and its response to treatment are based on data from the literature. In cases where no data is available, parameter values were obtained by fitting (in a least squares sense) time-courses of cell numbers to data from [1] wherein IGROV-1 xenografts established in mice were left untreated (control) or treated periodically with a fixed dose of carboplatin or ABT-737 (or both) for 4 weeks. Briefly, the model was reduced to represent the different *in vivo* experimental systems: no therapy (control), ABT-737-only therapy, carboplatin-only therapy, and combined therapies involving carboplatin and ABT-737. The fits were carried out using the computing software Matlab's in-built nonlinear, curve-fitting tool, "lsqcurvefit". Care was taken to fit no more than 2-3 parameters to any given set of experimental data. Biologically realistic values were chosen for parameters for which no experimental data was available. The model equations for each case and the parameters being fit are reported below.

S5.1 No Therapy (Control) and ABT-737-only Therapy

The basic proliferation rate of IGROV-1 cells (λ_N), the carrying capacity of their microenvironment (K) and the Bax-dependent death rate of proliferating cells (r_X) are fixed by simultaneously fitting time-courses of simulated cell numbers to tumor xenograft growth data taken from [1], wherein IGROV-1 xenografts established in mice were left untreated (control) or treated daily with a fixed dose (100 mg/kg) of ABT-737 administered intraperitoneally for 4 weeks, and tumor size recorded at regular intervals. The following equations for xenograft growth and treatment with ABT-737 are obtained from

the full model system by setting the carboplatin concentrations $C_{perit} = C_P = C_T = 0$.

$$\frac{dN}{dt} = \lambda_N\, N \left(1 - \frac{N}{K}\right) - r_X\, X\, N, \tag{S5.1}$$

$$A_{perit}(t) = \alpha^*_{perit}\, e^{-\mu_A\, rem(t,\tau_A)}, \tag{S5.2}$$

$$\frac{dA_P}{dt} = \mu_A\, A_{perit} - K_A\, A_P, \tag{S5.3}$$

$$B(A_C) = \frac{-(\zeta_1\, \beta\, A_C + \zeta_2\, \alpha_C\, \beta) + \zeta_1\, \beta\, \sqrt{A_C^2 + \rho_1\, \alpha_C\, A_C + \rho_2\, \alpha_C^2}}{\bar{\nu}_1\, A_C + \alpha_C\, \bar{\nu}_2}, \tag{S5.4}$$

$$X(A_C) = -(\chi\, \sigma_1\, A_C + \chi\, \alpha_C\, \sigma_2) + \chi\, \sigma_1\, \sqrt{A_C^2 + \rho_1\, \alpha_C\, A_C + \rho_2\, \alpha_C^2}, \tag{S5.5}$$

$$\frac{dA_C}{dt} = \left(K^A_{PC}\, A_P - K^A_{CP}\, A_C\right) \frac{\alpha_P}{\alpha_P - \alpha_C\,(dB/dA_C) + \alpha_P\,(dX/dA_C)}, \tag{S5.6}$$

where

$$\alpha^*_{perit} = \begin{cases} 0, & \text{no therapy,} \\ \alpha_{perit}, & \text{ABT-737 therapy.} \end{cases} \tag{S5.7}$$

The fits that minimize in a least squares sense the residual between simulated and experimental data are presented in Figure 1C of the main text. The values of λ_N, K and r_X thus obtained are reported in Table S4.

S5.2 Carboplatin-only and Carboplatin + ABT-737 Combination Therapy

Next, the maximal rate of proliferating cell become growth arrested in response to carboplatin (r_C), the carboplatin-dependent rate at which arrested cells die (ρ_C) and the sensitivity of the arrested cells to changes in Bax (ρ_S) are fixed by simultaneously fitting simulated time-courses of cell numbers to tumor xenograft growth inhibition data taken from [1], wherein IGROV-1 xenografts established in mice were

treated weekly with a fixed bolus dose (30 mg/kg) of carboplatin alone or in combination with a fixed daily dose (100 mg/kg) of ABT-737 administered intraperitoneally for 4 weeks, and tumor size recorded at regular intervals. The equations for xenograft treatment with carboplatin alone or in combination with ABT-737 are:

$$\frac{dN}{dt} = \lambda_N N \left(1 - \frac{N + \int_{t-a_r}^{t} M(t,a)da}{K}\right) - r_X X N - \alpha_C(C_T) N + M(t, a = a_r), \tag{S5.8}$$

$$\frac{\partial M}{\partial t} + \frac{\partial M}{\partial a} = -\left(\rho_C + \rho_S r_X (X - X_{phys})\right) C_T(t-a) H(a - a_{char}) M, \tag{S5.9}$$

$$A_{perit}(t) = \alpha^*_{perit} e^{-\mu_A rem(t,\tau_A)}, \tag{S5.10}$$

$$\frac{dA_P}{dt} = \mu_A A_{perit} - \lambda_A A_P, \tag{S5.11}$$

$$B(A_C) = \frac{-(\zeta_1 \beta A_C + \zeta_2 \alpha_C \beta) + \zeta_1 \beta \sqrt{A_C^2 + \rho_1 \alpha_C A_C + \rho_2 \alpha_C^2}}{\bar{\nu}_1 A_C + \alpha_C \bar{\nu}_2}, \tag{S5.12}$$

$$X(A_C) = -(\chi \sigma_1 A_C + \chi \alpha_C \sigma_2) + \chi \sigma_1 \sqrt{A_C^2 + \rho_1 \alpha_C A_C + \rho_2 \alpha_C^2}, \tag{S5.13}$$

$$\frac{dA_C}{dt} = \left(K^A_{PT} A_P - K^A_{TP} A_C\right) \frac{\alpha_P}{\alpha_P - \alpha_C (dB/dA_C) + \alpha_P (dX/dA_C)}, \tag{S5.14}$$

$$C_{perit}(t) = C_{perit}(t=0) e^{-\mu_C rem(t,\tau_C)}, \tag{S5.15}$$

$$\frac{dC_P}{dt} = \mu_C \frac{V^C_{perit}}{V^C_P} C_{perit} - K_C C_P - K^C_{PT} C_P + K_{TP} \frac{V^C_T}{V^C_P} C_T, \tag{S5.16}$$

$$\frac{dC_T}{dt} = K^C_{PT} \frac{V^C_P}{V^C_T} C_P - K^C_{TP} C_T, \tag{S5.17}$$

where

$$\alpha_C(C_T) = r_C \frac{C_T^2}{C_T^2 + K_{carb}^2}, \qquad \text{(S5.18)}$$

$$M(t, 0) = \alpha_C(C_T)\, N, \qquad \text{(S5.19)}$$

$$\alpha^*_{perit} = \begin{cases} 0, & \text{carboplatin-only therapy,} \\ \alpha_{perit}, & \text{combination therapy,} \end{cases} \qquad \text{(S5.20)}$$

and where $X_{phys} = 27.83$ nM is the intracellular concentration of free Bax in the absence of ABT-737 therapy (see section S3.2 above). The fits that minimize in a least squares sense the residual between simulated and experimental data are presented in Figure 1D of the main text. The values of r_C, ρ_C and ρ_S thus obtained are reported in Table S4.

S5.3 Parameter Sensitivity

A sensitivity analysis was carried out by varying the parameters associated with the cellular response to carboplatin and ABT-737 that were previously estimated from experimental data. These include: the rate at which proliferating cells die (r_X, see equation (S1.14)); the concentration of carboplatin at which the rate of proliferating cell arrest is half its maximum value (K_{carb}) and the maximum rate at which proliferating cells become growth arrested in response to carboplatin (r_C, see equation (S1.15)); and the rate at which arrested cells die in response to carboplatin-induced DNA damage (ρ_C) and the sensitivity of arrested cells to intracellular Bax (ρ_S, see equation (S1.16)). In each case, the residual between simulated and experimental data is computed as each parameter is varied from its baseline estimate, and the resulting percentage increase in this residual is plotted as a function of the change in the value of parameter being varied.

Figures S4A,B reveal that the model predictions of tumor cell numbers are highly sensitive to r_X and α_C. A 10% change over the baseline value of r_X increases the residual in the fits shown in Figure 1C

by over 20% (Figure S4A), while a 14% change in α_C has the same effect on the residual in these fits (Figure S4B). From Figure S4D, we observe that sensitivity to changes in ρ_C is less marked, with a 20% change in ρ_C over its baseline value predicted to result in a 20% increase in the residual of the fits shown in Figure 1D. The model is more robust to changes in K_{carb}: Figure S4C reveals that a change in K_{carb} of at least 50% over its baseline value is required to induce a 20% increase in the residual in the fits shown in Figure 1D. Model predictions are also robust to changes in ρ_S (see Figure S4E). However, for very small values of ρ_S, the residual in the fits shown in Figure 1D increases rapidly.

Finally, we investigate the effect of varying the carboplatin infusion time T_i on tumor xenograft size at the end of 4 weeks of treatment with carboplatin alone, fixing its weekly dose at 30 mg/kg, as in [1]. Figure S4F shows how the numbers of proliferating and arrested cells averaged over the period of carboplatin administration change as T_i is varied from 0 (bolus) to 5 days. An infusion time of between 1 and 2 hours is predicted to maximize tumor growth inhibition. Increasing T_i above 2 hours results in a rapid decrease in the level of tumor growth inhibition, with infusions lasting 2 or more days having almost no effect on final xenograft sizes. This is because slow infusions of carboplatin lead to low tissue concentrations of the drug, so that the level of DNA damage induced in proliferating cells is minimal. Interestingly, infusions lasting 8 hours are predicted to give the highest fraction of cells in a growth arrested state. This is possibly due to a high rate of proliferating cell arrest coupled with a relatively low rate of arrested cell death. We remark that since arrested cells have previously been shown to be highly sensitive to intracellular Bcl-xL levels [8], when carboplatin is given with ABT-737, a carboplatin infusion time of 8 hours is expected maximally to exploit the synergy between the two drugs. Figures 3, 4 and 5 of the main text corroborate this prediction.

From the above discussion, we expect that the optimal protocols predicted by our model will be relatively robust to changes in the parameters ρ_C, K_{carb} and ρ_S and highly sensitive to variations in r_X, α_C or T_i.

References

[1] Witham J, Valenti MR, De-Haven-Brandon AK, Vidot S, Eccles SA, et al. (2007) The Bcl-2/Bcl-xL family inhibitor ABT-737 sensitizes ovarian cancer cells to carboplatin. Clin Cancer Res 13: 7191-7198.

[2] Bajaj G, Yeo Y (2010) Drug delivery systems for intraperitoneal therapy. Pharm Res 27: 735-738.

[3] Lukas G, Brindle SD, Greengard P (1971) The route of absorption of intraperitoneally administered compounds. J Pharmacol Exp Ther 178: 562-564.

[4] Chen C, Cui J, Zhang W, Shen P (2007) Robustness analysis identifies the plausible model of the Bcl-2 apoptotic switch. FEBS Lett 158: 5143-5150.

[5] Hua F, Cornejo MG, Cardone MH, Stokes CL, Lauffenburger DA (2005) Effects of Bcl-2 levels on Fas signaling-induced caspase-3 activation: molecular genetic tests of computational model predictions. J Immunol 175: 985-995.

[6] Siddik ZH, Newell DR, Boxall FE, Harrap KR (1987) The comparative pharmacokinetics of carboplatin and cisplatin in mice and rats. Biochem Pharmacol 36: 1925-1932.

[7] Jain HV, Byrne HM (2012) Qualitative analysis of an integro-differential equation model of periodic chemotherapy. Appl Math Lett 25: 2132-2136.

[8] Jain HV, Meyer-Hermann M (2011) The molecular basis of synergism between carboplatin and ABT-737 therapy targeting ovarian carcinomas. Cancer Res 71: 705-715.

[9] Tse C, Shoemaker AR, Adickes J, Anderson MG, Chen J, et al. (2005) ABT-263: a potent and orally bioavailable Bcl-2 family inhibitor. Cancer Res 68: 3421-3428.

[10] Oltersdorf T, Elmore SW, Shoemaker AR, Armstrong RC, Augeri DJ, et al. (2005) An inhibitor of Bcl-2 family proteins induces regression of solid tumours. Nature 435: 677-681.
