# Supplementary material for: Exploiting the Synergy between Carboplatin and ABT-737 in the Treatment of Ovarian Carcinomas
Source: PLoS One. 2014 Jan 6;9(1):e81582. doi: 10.1371/journal.pone.0081582 (PMC3882219; doi:10.1371/journal.pone.0081582)
Supplement: Table S1 — List of parameter values relating to ABT-737 pharmacokinetics and pharmacodynamics. (PDF) [file pone.0081582.s006.pdf]

Table S1: List of parameter values relating to ABT-737 pharmacokinetics and pharmacodynamics.

Parameter	Value	Source
τ_A	1 day	[1]
α_{perit}	11.56×10^3 nM	[2]
μ_A	6 per day	see text
λ_A	3.37 per day	[2]
K_{CP}^A	2.40 per day	see text
K_{PC}^A	3×10^{-3} per day	see text
K_A	3.36 per day	see text
k_a^A	172.80 per day per nM	[3,4][1]
k_d^A	172.80 per day	[4][2]
k_a^X	172.80 per day per nM	[3]
k_d^X	1728 per day	[3]
β	75 nM	[3]
χ	83.33 nM	[3]

References

[1] Witham J, Valenti MR, De-Haven-Brandon AK, Vidot S, Eccles SA, et al. (2007) The Bcl-2/Bcl-xL family inhibitor ABT-737 sensitizes ovarian cancer cells to carboplatin. Clin Cancer Res 13: 7191-7198.

[2] Tse C, Shoemaker AR, Adickes J, Anderson MG, Chen J, et al. (2005) ABT-263: a potent and orally bioavailable Bcl-2 family inhibitor. Cancer Res 68: 3421-3428.

[3] Hua F, Cornejo MG, Cardone MH, Stokes CL, Lauffenburger DA (2005) Effects of Bcl-2 levels on Fas signaling-induced caspase-3 activation: molecular genetic tests of computational model predictions. J Immunol 175: 985-995.

[4] Oltersdorf T, Elmore SW, Shoemaker AR, Armstrong RC, Augeri DJ, et al. (2005) An inhibitor of Bcl-2 family proteins induces regression of solid tumours. Nature 435: 677-681.

[1] It is assumed that rate of the forward reaction of ABT-737 with Bcl-2/xL is similar to that of Bax binding to Bcl-2.
[2] The dissociation constant for ABT-737 binding Bcl-2/xL is ~ 1 nM.
