# Supplementary material for: Exploiting the Synergy between Carboplatin and ABT-737 in the Treatment of Ovarian Carcinomas
Source: PLoS One. 2014 Jan 6;9(1):e81582. doi: 10.1371/journal.pone.0081582 (PMC3882219; doi:10.1371/journal.pone.0081582)
Supplement: Table S2 — List of non-dimensional parameter values relating to ABT-737 pharmacokinetics and pharmacodynamics. (PDF) [file pone.0081582.s007.pdf]

Table S2: List of non-dimensional parameter values relating to ABT-737 pharmacokinetics and pharmacodynamics.

Parameter	Expression	Value
$\bar{\mu}_A$	$\tau_A\,\mu_A$	6
$\bar{\eta}_A$	$\mu_A\,\tau_A\,\alpha_{perit}/\alpha_P$	11.66
$\bar{K}_A$	$K_A\,\tau_A$	3.36
ϵ	$1/(k_d^X\,\tau_A)$	5.79×10^{-4}
$\bar{\kappa}_0$	$k_d^X\,\tau_A\,\alpha_C/\alpha_P$	1.30
$\bar{\kappa}_1$	$K_{PC}^A\,\tau_A\,\alpha_P/\alpha_C$	3.96
$\bar{\kappa}_2$	$K_{CP}^A\,\tau_A$	2.40
$\bar{\gamma}_1$	β/α_C	16.70
$\bar{\gamma}_2$	β/χ	0.90
$\bar{\nu}_1$	$k_a^A\,\alpha_T/k_d^X$	0.45
$\bar{\nu}_2$	k_d^A/k_d^X	0.10
$\bar{\nu}_3$	$k_a^X\,\chi/k_d^X$	8.33
ζ_1	$\bar{\nu}_1/(2\bar{\gamma}_2\bar{\nu}_3)$	0.03
ζ_2	$\bar{\nu}_2(1+\bar{\nu}_3-\bar{\nu}_3\bar{\gamma}_2)/(2\,\bar{\gamma}_2\bar{\nu}_3)$	0.01
σ_1	$\bar{\nu}_1/(2\bar{\nu}_2\bar{\nu}_3)$	0.27
σ_2	$(1-\bar{\nu}_3+\bar{\nu}_3\bar{\gamma}_2)/(2\,\bar{\nu}_3)$	0.01
ρ_1	$2\,\bar{\nu}_2(1+\bar{\nu}_3+\bar{\nu}_3\bar{\gamma}_2)/\bar{\nu}_1$	7.50
ρ_2	$\bar{\nu}_2^2\left[1+2\,\bar{\nu}_3(1+\bar{\gamma}_2)+\bar{\nu}_3^2(1-\bar{\gamma}_2)^2\right]/\bar{\nu}_1^2$	1.65
