# Supplementary material for: Exploiting the Synergy between Carboplatin and ABT-737 in the Treatment of Ovarian Carcinomas
Source: PLoS One. 2014 Jan 6;9(1):e81582. doi: 10.1371/journal.pone.0081582 (PMC3882219; doi:10.1371/journal.pone.0081582)
Supplement: Table S3 — List of parameter values relating to carboplatin pharmacokinetics. (PDF) [file pone.0081582.s008.pdf]

Table S3: List of parameter values relating to carboplatin pharmacokinetics.

Parameter	Value	Source
w_C	371.25 Da	pubchem database
μ_C	201.60 per day	see text
K_{PT}^{C}	201.60 per day	[1]
K_{TP}^{C}	201.60 per day	[1]
K_C	90.72 per day	[1]
V_{perit}^{C}	2.50×10^{-3} L	see text
V_P^C	2.50×10^{-3} L	[1]
V_T^C	3.96×10^{-3} L	[1]

References

[1] Siddik ZH, Newell DR, Boxall FE, Harrap KR (1987) The comparative pharmacokinetics of carboplatin and cisplatin in mice and rats. Biochem Pharmacol 36: 1925-1932.
