# Supplementary material for: Exploiting the Synergy between Carboplatin and ABT-737 in the Treatment of Ovarian Carcinomas
Source: PLoS One. 2014 Jan 6;9(1):e81582. doi: 10.1371/journal.pone.0081582 (PMC3882219; doi:10.1371/journal.pone.0081582)
Supplement: Table S4 — List of parameter values relating to xenograft growth and treatment. (PDF) [file pone.0081582.s009.pdf]

Table S4: List of parameter values relating to xenograft growth and treatment.

Parameter	Value	Source
λ_N	0.13 per day	[1]
λ_R	0.13 per day	[1]
K	18.18 cells (in millions)	[1]
r_X	1.53×10^{-3} per nM Bax per day	[1]
r_C	5.47 per day	[1]
K_{carb}	21 μM	[2]
ρ_C	8.21×10^{-3} per μM carboplatin per day	[1]
ρ_S	134 per μM carboplatin	[1]
α_{perit} (corresponding to 100 mg/kg ABT-737)	11.56 μM	[1]
κ_{perit} (corresponding to 30 mg/kg carboplatin)	500.36 μM	[1]
X_{phys}	27.83 nM	[3]

<u>References</u>

[1] Witham J, Valenti MR, De-Haven-Brandon AK, Vidot S, Eccles SA, et al. (2007) The Bcl-2/Bcl-xL family inhibitor ABT-737 sensitizes ovarian cancer cells to carboplatin. Clin Cancer Res 13: 7191-7198.

[2] Jain HV, Meyer-Hermann M (2011) The molecular basis of synergism between carboplatin and ABT-737 therapy targeting ovarian carcinomas. Cancer Res 71: 705-715.

[3] Hua F, Cornejo MG, Cardone MH, Stokes CL, Lauffenburger DA (2005) Effects of Bcl-2 levels on Fas signaling-induced caspase-3 activation: molecular genetic tests of computational model predictions. J Immunol 175: 985-995.

[1] For simplicity, the basic proliferation rates of carboplatin-sensitive (N) and carboplatin-resistant (R) cells are taken to be equal.
